# PLD1 and PLD2 promote an immunosuppressive tumor microenvironment via CCL19-dependent macrophage polarization and PD-L1 induction

**DOI:** 10.1038/s12276-026-01742-y

**Published:** 2026-06-04

**Authors:** Hyesung Lee, Seong Hun Lim, Won Chan Hwang, Tae Hyun Kim, Kyeongseok Bae, Jinu Lee, Do Sik Min

**Affiliations:** 1 College of Pharmacy, Yonsei University, Incheon, Republic of Korea; 2 Department of Biological Sciences, Ulsan National Institute of Science and Technology, Ulsan, Republic of Korea

**Keywords:** Melanoma, Cancer microenvironment

## Abstract

Tumor cells shape the immunosuppressive tumor microenvironment (TME) through coordinated interactions with tumor-associated macrophages (TAMs), regulatory T cells (T_regs_), immune checkpoint pathways and suppressive cytokines, thereby limiting the efficacy of immunotherapy across diverse cancer types. Phospholipase D (PLD) enzymes, particularly the PLD1 and PLD2 isoforms, have been implicated in oncogenic signaling and tumor progression; however, their tumor-intrinsic roles in modulating the immune landscape remain largely undefined. Here we demonstrated that both genetic ablation and pharmacological inhibition of PLD1 and PLD2 reprogram the TME and enhance antitumor immunity in a syngeneic melanoma model. Elevated PLD expression is associated with increased infiltration of M2-like TAMs, decreased ‘eat me’ signals and enhanced ‘don’t eat me’ signals. Conversely, loss or inhibition of PLD1 and PLD2 reduced T_reg_ recruitment and enhanced infiltration of Th1, Th17 and cytotoxic CD8⁺ T cells, accompanied by downregulation of immune checkpoint molecules and restoration of T cell effector function. Depletion studies revealed that PLD-driven TAM polarization critically impairs CD8⁺ T cell-mediated antitumor responses. Mechanistically, PLD1 and PLD2 enhance CCL19 secretion, promote macrophage polarization toward an immunosuppressive phenotype and induce programmed death-ligand 1 (PD-L1) expression by activating the PI3K–Akt–NF-κB signaling axis, thereby promoting tumor immune evasion. Notably, PLD inhibition reduced CCL19 production, abrogated IFN-γ- or CCL19-induced PD-L1 expression, decreased TAM infiltration and increased CD8⁺ T cell infiltration, collectively shifting the TME toward an immune-activated state. These findings suggest that tumor-intrinsic PLD1 and PLD2 function as modulators of immune suppression and that PLD inhibition represents a promising strategy to overcome resistance to cancer immunotherapy.

## Targeting PLD enzymes boosts antitumor immunity in melanoma

This study explores the role of phospholipase D (PLD) isoforms, PLD1 and PLD2, in melanoma, focusing on their impact on the tumor microenvironment (TME). The authors reveal that PLD1/2 are upregulated in melanoma, promoting tumor growth and immune evasion by modulating macrophage polarization and PD-L1 expression. Using CRISPR–Cas9 knockout models and pharmacological inhibition, they demonstrate that PLD1/2 deficiency shifts macrophages from an M2-like to an M1-like phenotype, enhancing T cell-mediated antitumor immunity. PLD1/2 deficiency reduces secretion of CCL19, which functions as an upstream regulator of immunosuppressive macrophage polarization and PD-L1 expression via the PI3K–Akt–NF-κB pathway. The findings suggest that targeting PLD1/2 could enhance cancer immunotherapy by reprogramming the immune landscape, highlighting the potential of PLD inhibitors as therapeutic agents. Future research could explore the applicability of these findings across different cancer types and further elucidate the signaling pathways involved.

This summary was initially drafted using artificial intelligence, then revised and fact-checked by the author.

## Introduction

Cancer develops through interactions between malignant cells and the surrounding tumor microenvironment (TME), which comprises diverse stromal components, including multiple immune cell subsets, fibroblasts and endothelial cells^1–3^. Accumulating evidence indicates that the immunosuppressive TME drives tumor initiation, progression and therapeutic resistance^1^. Consequently, targeting the TME has emerged as a key strategy to reverse immunosuppression and enhance antitumor immune responses. Among immune populations within the TME, tumor-associated macrophages (TAMs) are vital in establishing an immunosuppressive milieu by promoting tumor growth, angiogenesis and immune evasion through continuous crosstalk with cancer cells^2–4^. TAMs exhibit substantial phenotypic plasticity, transitioning between pro-inflammatory and tumoricidal M1-like states, as well as anti-inflammatory and tumor-promoting M2-like states, in response to local microenvironmental cues^5^. The M1-like and M2-like nomenclature is used to denote dominant functional polarization states within the TME, rather than rigid or mutually exclusive macrophage subsets. M2-like TAMs function as major drivers of tumor progression and potent suppressors of adaptive immunity. Consequently, reprogramming TAMs from an M2-like immunosuppressive phenotype to a pro-inflammatory M1-like state is a promising strategy to enhance T cell-mediated immunity and overcome immune resistance^6^. Notably, tumor-PD-L1 and TAMs engage in a bidirectional regulatory loop in which PD-L1 modulates TAM polarization while TAM-derived cytokines promote PD-L1 expression in cancer cells, collectively amplifying immune evasion^7^.

Melanoma, the most aggressive form of skin cancer, accounts for most skin cancer-related deaths worldwide^8^. Although immune checkpoint blockade (ICB) has produced substantial clinical responses in advanced melanoma by restoring cytotoxic T cell activity, most patients exhibit primary resistance or develop secondary resistance during therapy^9–11^. Addressing this limitation requires a deeper understanding of the molecular and cellular mechanisms driving immune suppression within the melanoma TME.

PD-L1 is expressed in tumor cells and regulates immune responses through dual mechanisms. Through interaction with PD1 in T cells, PD-L1 induces T cell exhaustion or apoptosis and attenuates antitumor activity^12,13^. Beyond this direct inhibition, PD-L1 shapes the broader TME by promoting immunosuppressive populations such as TAMs and myeloid-derived suppressor cells (MDSCs), thereby reinforcing immune escape^14,15^. Tumor-derived cytokines and chemokines influence macrophage polarization and immune checkpoint expression, driving metastasis, angiogenesis and therapeutic resistance. Accordingly, identifying tumor-intrinsic regulators that control cytokine secretion, macrophage polarization and PD-L1 induction is critical to overcoming immune suppression in cancer^16,17^.

Recent findings have indicated that tumor-intrinsic lipid signaling pathways serve as key modulators of cytokine secretion and immune cell recruitment. Among these, phospholipase D (PLD) and its enzymatic product phosphatidic acid (PA) function as central mediators of oncogenic signaling, vesicular trafficking and membrane dynamics^18^. Although the canonical roles of PLD1 and PLD2 (PLD1/2) in promoting tumor proliferation, invasion and metastasis are well established^18,19^, their contributions to immunosuppressive TME remodeling remain poorly understood. Evidence suggests that PLD isoforms exert context-dependent immunomodulatory effects. Inhibition of PLD1 induces immunogenic cell death and enhances the efficacy of cancer immunotherapy in colorectal cancer^19^, whereas PLD2 regulates cytokine production in macrophages and modulates T cell responses^20^. These findings suggest that PLD isoforms influence antitumor immunity by regulating cytokine secretion and immune cell recruitment. However, the mechanisms by which tumor-intrinsic PLD1/2 coordinate macrophage polarization and immune checkpoint regulation in the melanoma TME remain unknown. Here, we aimed to elucidate the mechanisms by which tumor-intrinsic PLD1/2 regulate CCL19-dependent TAM polarization and PD-L1 induction and to determine the broader importance of PLD1/2 activity in establishing an immunosuppressive TME.

## Materials and Methods

### Human skin cancer TMA analysis

Paraffin-embedded human skin cancer tissue microarrays (TMAs; US BIOMAX) were subjected to immunohistochemistry (IHC) to evaluate PLD1, PLD2 and CCL19 expression. TMA sections were stained using the protocol described in the IHC section. Digital images were acquired using a Panoramic MIDI II slide scanner (3DHISTECH). Protein expression was quantified using semiquantitative H-score analysis based on staining intensity (0 = negative, 1 = weak, 2 = moderate, 3 = strong) and the percentage of positive cells, using the formula^21^

H-score = (1 × % weakly positive cells) + (2 × % moderately positive cells) + (3 × % strongly positive cells) (range of 0–300).

All slides were evaluated by three independent observers blinded to sample identity.

### Generation of *Pld1*-KO and *Pld2*-KO B16F10 cell lines

Mouse B16F10 melanoma cells (B16-OVA, RRID:CVCL_0159; American Type Culture Collection (ATCC)) were maintained in Dulbecco’s modified Eagle medium (DMEM) supplemented with 10% fetal bovine serum (FBS) at 37 °C in 5% CO_2_. *Pld1*-KO (*Pld1*-KO) and *Pld2*-KO (*Pld2*-KO) cell lines were generated via lentiviral CRISPR–Cas9 editing. Single-guide (sg)RNAs targeting the PX domain of each PLD isoform were designed using the Broad Institute GPP tool and cloned into a lentiviral CRISPR vector. The sgRNA sequences were 5′-CACGATCGAGTTAACGCACG-3′ for *Pld1* and 5′-GATAGAAAATCTCGACACCC-3′ for *Pld2*. B16F10 cells were transduced with sgRNA-expressing lentiviruses, and KO clones were selected and expanded. All cell lines were routinely screened for *Mycoplasma* contamination using the Myco-Read Detection Kit (BIOMAX). In this study, the notation ‘*Pld1/2*-KO’ is used as a collective shorthand to denote either *Pld1*-KO or *Pld2*-KO single-KO cells.

### Cell proliferation and clonogenic assays

For proliferation assays, 1 × 104 cells were seeded into 96-well plates (100 μl per well; three replicates per condition). Cell growth was monitored for 48 h using the IncuCyte Zoom live-cell imaging system (Essen BioScience), and confluence was quantified using IncuCyte software. For clonogenic assays, 1,000 cells were seeded in six-well plates (three wells per group) and cultured in complete medium; media were replaced every 3 days. After 10 days, colonies were washed with PBS, fixed in 70% ethanol for 5 min at room temperature and stained with 0.5% crystal violet in 25% methanol for 15 min. Plates were rinsed and air-dried and colonies were counted.

### Cell cycle assay

Cell cycle distribution was analyzed via flow cytometry using bromodeoxyuridine (BrdU) incorporation and DNA content staining. Cells were pulse-labeled with 10 μM BrdU for 2 h at 37 °C, fixed and permeabilized according to the BD Pharmingen FITC BrdU Flow Kit protocol and stained with FITC-anti-BrdU and 7-aminoactinomycin D (7-AAD). Samples were acquired using a BD FACSAria III and analyzed with FlowJo v10 to determine the proportions of cells in G_0_/G_1_, S and G_2_/M phases.

### Wound healing and Transwell migration and invasion assays

For wound healing assays, 1 × 10^5^ cells were seeded in 24-well plates and cultured in DMEM with 10% FBS to approximately 70% confluence. A linear scratch was generated using a 200-μl pipette tip; debris was removed by washing with PBS, and cells were maintained in serum-free DMEM. Wound closure was imaged at 0, 24 and 48 h, and the remaining wound area was quantified to calculate the closure percentage.

For Transwell migration assays, 2 × 10^5^ cells in serum-free DMEM was placed in the upper chamber of 8-μm pore inserts (SPL Life Science), and DMEM containing 10% FBS was added to the lower chamber as a chemoattractant. After 24 h at 37 °C, nonmigrated cells were removed and migrated cells on the underside of the membrane were fixed in 4% paraformaldehyde, permeabilized with methanol, stained with 0.5% crystal violet and counted in five random fields.

For invasion assays, Transwell inserts were coated with growth factor-reduced Matrigel (Corning) and polymerized for 1 h at 37 °C. Approximately 2 × 10^5^ cells were seeded into the coated upper chambers in serum-free medium, and 10% FBS-containing medium was added to the lower chambers. After 24 h, invading cells were fixed, stained and quantified as in the migration assay.

### Mouse tumor models and in vivo treatment protocols

All animal procedures were approved by the Yonsei University Institutional Animal Care and Use Committee (IACUC-202007-1113-01) and conducted in accordance with institutional guidelines. Male C57BL/6 mice (6–8-weeks old, Orient Bio) were maintained under specific pathogen-free conditions and randomly assigned to experimental groups (*n* = 5–7 per group). Investigators were blinded to group assignments during tumor measurements. Wild-type (WT) or *Pld1*- or *Pld2*-deficient B16F10 melanoma cells (2 × 10^5^ in 50 μl PBS) were injected subcutaneously into the right flank. Tumor dimensions were measured every 2 days, and tumor volume was calculated as TV = (*W* × *L*^2^)/2, where *W* denotes width and *L* denotes length. Mice were killed by CO_2_ asphyxiation at predefined endpoints for immune profiling and histological analyses.

For macrophage depletion, mice were intraperitoneally administered antimouse colony-stimulating factor 1 receptor (CSF1R) antibody (Clone AFS98, BioXcell; 100 μg per mouse, i.p.) every 2 days starting the day after tumor inoculation; isotype IgG was used as positive control. For T cell subset depletion, anti-CD4 or anti-CD8α antibodies (Clone GK1.5, BioXcell; 100 μg per mouse, i.p.) were administered every 2 days beginning 7 days after implantation, and matched IgG was used as controls. PLD1 and PLD2 inhibition was performed by i.p. injection of the dual PLD1/2 inhibitor VU0155056 (Sigma-Aldrich) every 2 days starting 1 week after tumor inoculation.

### Tumor-infiltrating immune cell isolation and flow cytometry

Tumors were collected at experimental endpoints, minced and digested in Roswell Park Memorial Institute medium (RPMI)-1640 containing Liberase TL (20 μg/ml; Roche), Liberase DL (20 μg/ml; Roche) and DNase I (20 μg/ml; Sigma-Aldrich) for 1 h at 37 °C with gentle agitation. Digestion was quenched with 2 mM EDTA, and single-cell suspensions were prepared by filtering through a 40-μm strainer. Red blood cells (RBCs) were removed using RBC lysis buffer, and tumor-infiltrating leukocytes were enriched via Percoll gradient centrifugation. Immune cell subsets, including MDSCs, macrophages and CD4^+^/CD8+ T cells, were analyzed using flow cytometry. Antibody panels and gating strategies are presented in Supplementary Table 1 and Supplementary Figs. 2–4 and 7. In brief, after exclusion of doublets and dead cells, MDSCs were identified within CD45^+^ leukocytes and subdivided into monocytic MDSCs (M-MDSCs; Ly6C^+^Ly6G^−^) and polymorphonuclear MDSCs (PMN-MDSCs; Ly6C^−^Ly6G^+^). TAMs were defined as CD45^+^CD11b^+^F4/80^+^ cells following exclusion of CD11c^+^, TCRβ^+^ and Ly6G^+^ populations. T cells were gated as CD45^+^TCRβ^+^CD25^+^ cells and further separated into CD4^+^ and CD8^+^ subsets.

### In vitro phagocytosis assay

Macrophage-mediated phagocytosis was evaluated using RAW264.7 cells stimulated with LPS (20 ng/ml) for 24 h to induce an M1-like phenotype. B16F10 cells were labeled with 1 μM carboxyfluorescein succinimidyl ester (CFSE) for 20 min at 37 °C and washed before co-culture. CFSE-labeled tumor cells were added to RAW264.7 cells at a 1:2 macrophage-to-tumor cell ratio and co-incubated for 4 h at 37 °C. The cells were collected and stained with PE-anti-CD11b to identify macrophages. Phagocytosis was quantified using flow cytometry as the percentage of CD11b^+^CFSE^+^ macrophages. At least 10,000 events per sample were acquired on a BD FACSAria III and analyzed using FlowJo.

### Splenocyte isolation and ex vivo T cell restimulation

Spleens from tumor-bearing mice were processed into single-cell suspensions, subjected to RBC lysis and resuspended in RPMI-1640 with 10% FBS. For antigen-specific restimulation, 5 × 10^5^ splenocytes were plated in 48-well plates and cultured with or without OVA_257–264_ peptide (SIINFEKL, 1 μg/ml; Sigma-Aldrich) for 36 h. Brefeldin A (GolgiStop, BD Biosciences) was added during the final 12 h to block cytokine secretion. Cells were stained for CD4 and CD8 surface markers and for intracellular cytokines or effector molecules following fixation and permeabilization. Samples were acquired on a BD FACSAria III and analyzed using FlowJo.

### IHC and IF

At study endpoints, tumors were collected, fixed in 4% paraformaldehyde overnight at 4 °C, transferred to 70% ethanol, embedded in paraffin and sectioned at 5 μm. Sections were deparaffinized, rehydrated and treated with 0.3% hydrogen peroxide for 30 min to block endogenous peroxidase activity. Antigen retrieval was performed using 10 mM citrate buffer (pH 6.0) at 95 °C for 15 min. Slides were incubated with primary antibodies (Supplementary Table 2) overnight at 4 °C, followed by incubation with species-appropriate secondary antibodies and detection using the VECTASTAIN Elite ABC kit with DAB substrate. Nuclei were counterstained with hematoxylin. Whole-slide images were acquired using a Panoramic MIDI II scanner, and staining intensity or positive cell counts were quantified via masked evaluation. For immunofluorescence (IF) staining, antigen-retrieved paraffin sections were incubated with primary antibodies overnight at 4 °C, followed by fluorophore-conjugated secondary antibodies for 1 h at room temperature in the dark. Slides were mounted using antifade medium, and fluorescent images were acquired using a Zeiss LSM confocal microscope under identical imaging settings across samples. For quantitative analysis of both IHC and IF data, at least three tumor sections per mouse and three randomly selected fields per section were analyzed. Tumors from three mice per experimental group were included in the analysis, and data were averaged per mouse, with statistical comparisons performed using the individual mouse as the unit of analysis without pooling fields across animals.

### CM collection and cytokine array analysis

Conditioned media (CM) were collected from WT, *Pld1*-KO and *Pld2*-KO B16F10 melanoma cells to evaluate tumor cell-derived secreted factors. Cells were cultured in serum-free DMEM for 48 h after reaching 70–80% confluence, and cell-free supernatants were collected and clarified by centrifugation. Cytokine levels in CM were analyzed using a mouse cytokine antibody array (Abcam, ab193659) according to the manufacturer’s protocol. Chemiluminescent signals were captured using a digital imaging system, and spot intensities were quantified using ImageJ. For each cytokine, signal intensity was normalized to internal positive controls and compared across groups.

### RNA extraction and real-time RT–PCR

Total RNA was extracted from cells using easy-BLUE reagent (Intron Biotechnology), and 5 μg RNA was reverse-transcribed into cDNA using the PrimeScript First Strand cDNA Synthesis Kit (Takara). Quantitative real-time PCR (qRT–PCR) was performed using a CFX96 instrument (Bio-Rad) with SYBR Green (Enzynomics). Gene expression was normalized to that of *Actb* and calculated using the 2^–ΔΔCt^ method. Primer sequences are presented in Supplementary Table 3.

### Western blotting

Cells were lysed in Passive Lysis Buffer (Promega) containing protease and phosphatase inhibitor cocktails. Equal amounts of protein were resolved via SDS–polyacrylamide gel electrophoresis and transferred to polyvinylidene difluoride membranes (Millipore). Membranes were blocked with 5% skim milk and incubated with the indicated primary antibodies overnight at 4 °C, followed by horseradish peroxidase-conjugated secondary antibodies. Signals were developed using a horseradish peroxidase substrate (Millipore) and imaged with a ChemiDoc system (Bio-Rad). Detailed information on all antibodies used in this study is presented in Supplementary Table 4. A polyclonal antibody recognizing both PLD1 and PLD2 was used as previously described^22^.

### BMDM differentiation and polarization assays

Bone marrow-derived macrophages (BMDMs) were generated from 7-week-old C57BL/6 mice, as described previously^23^. Bone marrow cells were flushed from femurs and tibias and cultured in DMEM supplemented with 10% FBS and 10 ng/ml macrophage colony-stimulating factor (M-CSF). After 3 days, nonadherent cells were removed, fresh M-CSF-containing medium was added and cultures were maintained until day 7 to obtain mature BMDMs. For polarization assays, BMDMs were stimulated for 24 h with 50% (v/v) CM derived from WT or *Pld1/2-KO* B16F10 cells. To assess cytokine-specific effects, recombinant CXCL1, CXCL2, CCL19 or VEGF-D (50 ng/ml each), all purchased from MyBioSource, was added individually to *Pld1/2-KO* CM. Macrophage polarization was assessed using flow cytometry with M1 markers (CD80, CD86, MHC-II) and M2 markers (CD163, CD206) and through morphological analysis under light microscopy.

### CD4^+^ and CD8^+^ T cell isolation and co-culture with polarized macrophages

CD4^+^ and CD8^+^ T cells were isolated from spleens of naive C57BL/6 mice using magnetic-activated cell sorting kits (Miltenyi Biotec) and incubated in complete RPMI-1640 supplemented with 10% FBS. BMDMs were polarized using B16F10-derived CM as described earlier; in specified treatment groups, recombinant CCL19 (50 ng/ml) was added during the 24-h priming period to promote immunosuppressive macrophage polarization. Following priming, BMDMs were washed and co-cultured with isolated T cells in 0.4-μm Transwell inserts to permit soluble factor exchange without direct cell–cell contact. WT CM-primed or *Pld*-KO CM-primed BMDMs were seeded in the lower chamber, and 2 × 10⁵ CD4⁺ or CD8⁺ T cells were added to the upper insert. Co-cultures were maintained for 48 h, after which T cells were collected and analyzed via qRT–PCR. For CD4⁺ T cells, transcriptional marker expression of T cell subsets (T-bet (*Tbx21*) for Th1, RORγt (*Rorc*) for Th17 and forkhead box P3 (*Foxp3*) for T_regs_) was quantified. For CD8⁺ T cells, the expression of cytotoxic effector genes (granzyme B (*Gzmb*), perforin (*Prf1*), FasL (*Fasl*)) was assessed to determine macrophage-mediated modulation of T cell activation and differentiation.

### PLD activity assay

PLD enzymatic activity in cell lysates was measured using the Amplex Red PLD Assay Kit (Invitrogen). B16F10 cells were treated with recombinant CCL19 (50 ng/ml) for 24 h, lysed and 100 μg of total protein was incubated with assay reagents. Fluorescence (Ex/Em, 530/590 nm) was measured using a Tecan microplate reader and normalized to protein concentration. All reactions were performed in triplicate. For live-cell monitoring of PLD activation, PA production was visualized using an EGFP-tagged PA-binding domain (PABD) reporter^24^. B16F10 cells transfected with PABD–EGFP were stimulated with CCL19 (50 ng/ml), and time-lapse images were acquired using confocal microscopy (Zeiss LSM) for up to 1 h.

### Analysis of public cancer databases for co-expression

To contextualize experimental findings in human tumors, co-expression analyses were performed using publicly available datasets. Gene expression profiles from The Cancer Genome Atlas (TCGA; RRID:SCR_003193) and additional cohorts were accessed via TIMER2.0 and cBioPortal (RRID:SCR_014555). Associations among *PLD1*, *PLD2*, *CCL19* and PD-L1 (*CD274*) were evaluated in melanoma (TCGA-skin cutaneous melanoma) and other cancer types. TIMER2.0 and cBioPortal were used to compute gene–gene correlations and assess immune infiltration metrics. Pearson or Spearman correlation coefficients and corresponding *P* values were obtained to quantify relationships between PLD isoforms and immunosuppressive components. These in silico results were integrated with experimental data to support mechanistic interpretations.

### Statistical analysis

Unless otherwise indicated, experiments were performed with at least three independent biological replicates, and data are presented as mean ± s.d. Statistical significance between two groups was determined using unpaired two-tailed Student’s *t*-tests, and comparisons among multiple groups were analyzed using one-way analysis of variance with appropriate post hoc tests. Statistical significance was set at *P* < 0.05. Statistical analyses and graph generation were performed using GraphPad Prism 8 (GraphPad Software Inc; RRID:SCR_002798). When applicable, investigators were masked to group assignments during data interpretation (for example, histological scoring). All key findings were independently replicated to ensure reproducibility.

## Results

### PLD1 and PLD2 are upregulated and promote the oncogenic phenotype of melanoma

**Fig. 1:**
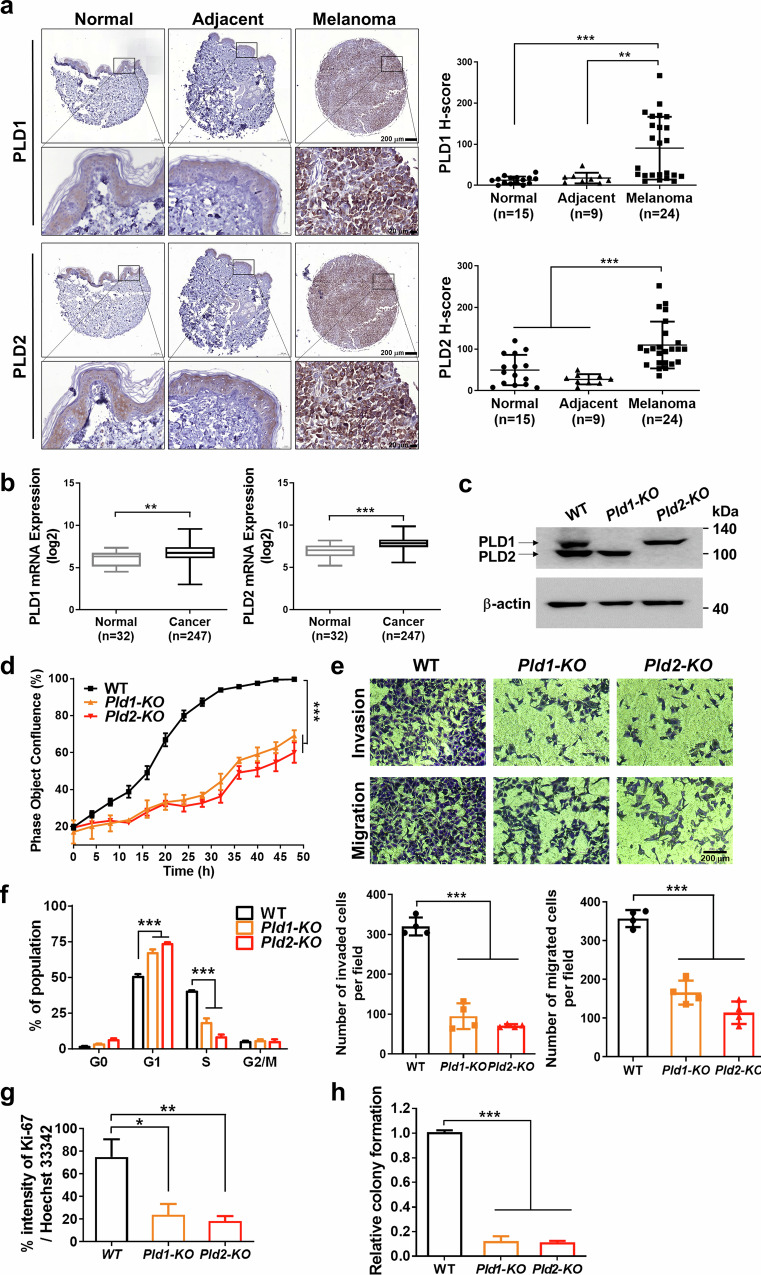
PLD1 and PLD2 are upregulated and promote the oncogenic phenotype of melanoma. **a** Representative IHC images showing PLD1 and PLD2 expression in a melanoma TMA with corresponding IHC score quantification. **b** Transcriptional levels of *PLD1* and *PLD2* in normal skin and melanoma cell lines obtained from the GENT2 database. **c** Immunoblot analysis of PLD1 and PLD2 expression in WT, *Pld1*-KO and *Pld2*-KO B16F10 cells. **d** Real-time proliferation of the indicated cell lines measured using the IncuCyte live-cell imaging system. **e** Representative images from Transwell invasion and migration assays with corresponding quantification. **f** Cell cycle distribution assessed using flow cytometry; percentages of cells in each phase are displayed. **g** Quantification of Ki-67⁺ nuclei, expressed as the percentage of Ki-67-positive cells relative to total Hoechst-stained nuclei. **h** Quantitative results from colony formation assays. Data are presented as mean ± s.e.m.; *P < 0.05; **P < 0.01; ***P < 0.001.

Given the established roles of PLD1/2 in tumorigenesis across multiple cancers^25^, we evaluated PLD1/2 expression in human skin cancer tissues using TMA. Immunohistochemical staining revealed substantially elevated PLD1 and PLD2 expression in melanoma compared with that in normal or adjacent nontumor skin (Fig. 1a). Similar upregulation was also observed in squamous cell carcinoma, basal cell carcinoma and metastatic lesions (Supplementary Fig. 1a). Consistent with these observations, transcriptomic profiling using the GENT2 database demonstrated significantly increased *PLD1* and *PLD2* mRNA levels in melanoma cell lines relative to that in normal skin cells (Fig. 1b). These findings indicate PLD1 and PLD2 overexpression in skin cancer with potential contributions to malignant progression.

CRISPR–Cas9-edited *Pld1*-deficient and *Pld2*-deficient B16F10 cells exhibited efficient isoform depletion, as confirmed using immunoblotting (Fig. 1c). Loss of either isoform significantly reduced melanoma cell proliferation (Fig. 1d), impaired migration and invasion (Fig. 1e) and delayed wound closure at 24 and 48 h (Supplementary Fig. 1b). Cell-cycle profiling revealed pronounced accumulation of *Pld1*-KO and *Pld2*-KO cells in G1 phase with reduced S-phase entry (Fig. 1f and Supplementary Fig. 1c). Consistent with this arrest, Ki-67 staining demonstrated reduced proliferative activity (Fig. 1g and Supplementary Fig. 1d), and colony formation assays showed substantial loss of clonogenic potential in *Pld1*/*2*-KO cells (Fig. 1h and Supplementary Fig. 1e). Collectively, these findings demonstrate PLD1 and PLD2 upregulation in melanoma and establish roles in tumor cell proliferation, migration, invasion and cell-cycle progression.

### Loss of PLD1 or PLD2 suppresses tumor growth by rewiring macrophage-driven **antitumor** immunity

**Fig. 2:**
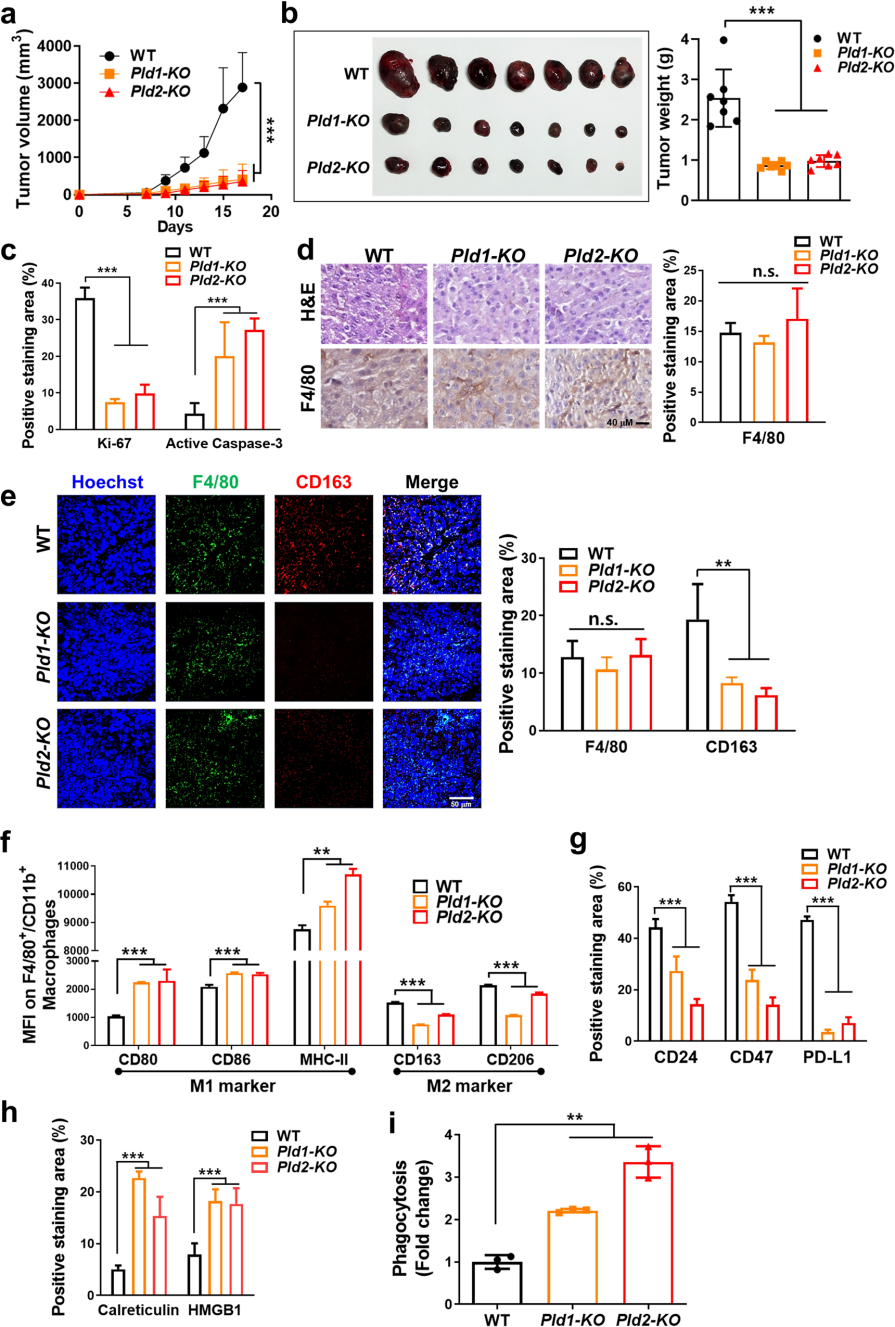
Loss of PLD1 and PLD2 suppresses tumor growth by rewiring macrophage-driven antitumor immunity. **a**, **b** WT and *Pld1/2*-KO B16F10 cells were subcutaneously injected into the flanks of C57BL/6 mice. Tumor volume (**a**) was monitored over 17 days, and tumor weight (**b**) was measured at the endpoint. **c** Quantification of Ki-67 and active caspase-3 in tumor sections. **d** Hematoxylin and eosin and IHC staining for F4/80 in tumor tissues. **e** Representative double-IF images showing F4/80 (green) and CD163 (red) with Hoechst 33342 (blue); bar graphs indicate relative fluorescence intensities. **f** Flow cytometry analysis of macrophage surface markers within F4/80^+^/CD11b^+^ tumor-infiltrating cells. **g**, **h** IHC analysis of phagocytosis-related checkpoint molecules in tumors derived from *Pld1/2*-KO cells, showing 'don't eat me' signals (CD24, CD47 and PD-L1) in **g** and 'eat me' signals (calreticulin and HMGB1) in **h**. **i** Flow cytometry analysis of phagocytosis by LPS-activated RAW264.7 macrophages co-cultured with WT or *Pld1/2*-KO B16F10 cells. Data are presented as mean ± s.e.m.; **P < 0.01; ***P < 0.001; n.s., not significant.

WT, *Pld1*-KO and *Pld2*-KO B16F10 cells were subcutaneously implanted into C57BL/6 mice to define in vivo roles of PLD1/2 in melanoma progression. Tumors lacking either PLD1 or PLD2 exhibited substantially reduced growth relative to that in WT tumors (Fig. 2a, b). Consistent with previous reports^26^, WT tumor-bearing mice developed pronounced splenomegaly, whereas spleen weights in *Pld1*/*2*-KO tumor-bearing mice were comparable to those of nontumor-bearing controls (Supplementary Fig. 2a). IHC analyses revealed decreased Ki-67 staining and increased active caspase-3 staining in *Pld1*/*2*-KO tumors, indicating suppressed proliferation and enhanced apoptosis (Fig. 2c and Supplementary Fig. 2b). Total abundance of F4/80⁺ macrophages and MDSCs remained comparable across groups (Fig. 2d and Supplementary Fig. 2c, d), whereas IF staining demonstrated a substantial reduction in CD163⁺F4/80⁺ M2-like macrophages within *Pld1*/*2*-KO tumors (Fig. 2e). Flow cytometry revealed increased expression of M1-associated markers (CD80, CD86, MHC-II) and decreased expression of M2 markers (CD163, CD206) in PLD1/2-deficient tumors (Fig. 2f and Supplementary Fig. 2e), indicating TAM reprogramming toward an M1-skewed phenotype.

We examined phagocytic signaling to evaluate functional consequences of this shift. ‘Don’t eat me’ cues, including CD24, CD47 and PD-L1, were substantially reduced, whereas ‘eat me’ signals such as calreticulin and HMGB1 were elevated in *Pld1*/*2*-KO tumors (Fig. 2g, h and Supplementary Fig. 2f, g). Correspondingly, LPS-activated RAW264.7 macrophages exhibited enhanced phagocytic uptake of *Pld1*/*2*-KO melanoma cells (Fig. 2i and Supplementary Fig. 2h). These findings demonstrate PLD1/2-dependent maintenance of M2-like TAM polarization with suppression of innate immune activation, whereas PLD1/2 loss promotes an M1-like, prophagocytic state that enhances antitumor immunity.

### Targeting PLD1/2 reprograms tumor immunity by enhancing CD8^+^ T cell cytotoxicity and Th1/Th17 responses

**Fig. 3:**
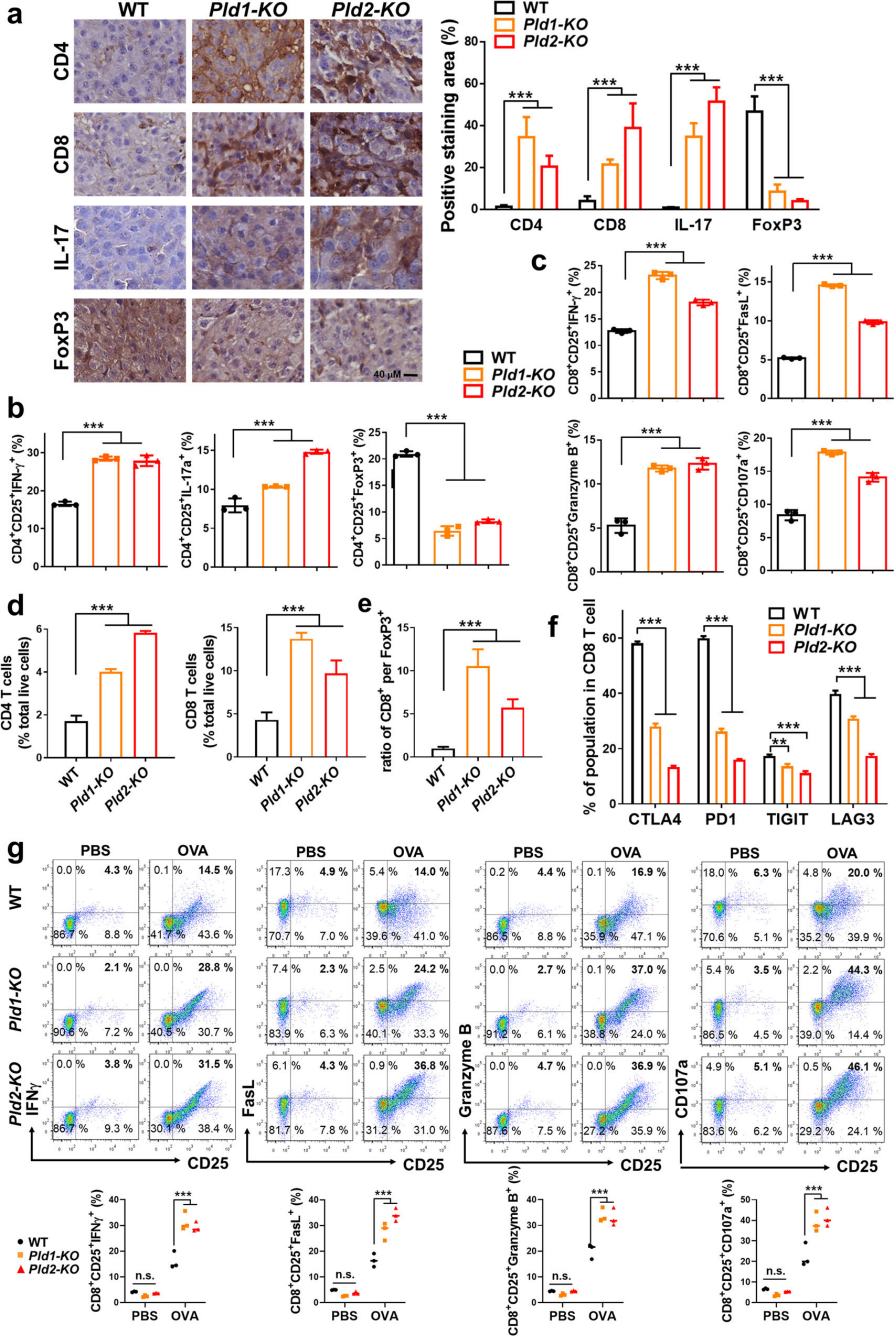
Targeting PLD1/2 reprograms tumor immunity by enhancing CD8^+^ T cell cytotoxicity and Th1/Th17 responses. **a** Representative IHC images and quantification of indicated markers in tumor sections. **b** Flow cytometric analysis of IFNγ^+^, IL-17a^+^ and FoxP3^+^ subsets within CD4^+^/CD25^+^ T cells. **c** Flow cytometric analysis of cytotoxic markers in CD8⁺/CD25⁺ T cells. **d**, **e** Quantification of CD4^+^ and CD8^+^ T cells (**d**) among live tumor-infiltrating lymphocytes and calculation of the CD8⁺ T cell-to-T_reg_ ratio (**e**). **f** Flow cytometric analysis of immune checkpoint expression in tumor-infiltrating CD8^+^/CD25^+^ T cells. **g** Ex vivo restimulation of splenic T cells from tumor-bearing mice with OVA peptide for 2 days, followed by flow cytometric assessment of CD8^+^/CD25^+^ T cells. Data are presented as mean ± s.e.m.; **P < 0.01; ***P < 0.001; n.s., not significant.

We analyzed tumor-infiltrating lymphocytes and systemic T cell responses using IHC and flow cytometry to define PLD1/2-mediated immune regulation. *Pld1*/*2*-KO tumors exhibited increased infiltration of CD4⁺, CD8⁺ and IL-17⁺ T cells with reduced FoxP3⁺ T_regs_ (Fig. 3a). Flow cytometry revealed expansion of Th1 (IFNγ⁺CD4⁺) and Th17 (IL-17A⁺CD4⁺) subsets with concomitant reduction of FoxP3⁺ T_regs_ (Fig. 3b and Supplementary Fig. 3a), indicating pro-inflammatory CD4⁺ T cell polarization. *Pld1*/*2*-KO tumors also contained increased proportions of cytotoxic CD8⁺ T cells expressing IFNγ, FasL, granzyme B and CD107a (Fig. 3c and Supplementary Fig. 3b). We also observed increased CD4⁺ and CD8⁺ T cell infiltration (Fig. 3d and Supplementary Fig. 3c) with elevated CD8⁺/FoxP3⁺ T_reg_ ratios (Fig. 3e). The expression of immune checkpoint molecules, including CTLA4, PD1, TIGIT and LAG3, was reduced in CD4⁺ and CD8⁺ T cells (Fig. 3f and Supplementary Fig. 3d), consistent with reduced T cell exhaustion within the TME.

We confirmed ovalbumin (OVA) expression in WT and *Pld1*/*2*-KO B16F10 cells (Supplementary Fig. 3e) and outlined the ex vivo restimulation workflow (Supplementary Fig. 3f). Splenic T cells isolated from tumor-bearing mice were stimulated with OVA peptide ex vivo. Activated CD8⁺ T cells from *Pld1*-KO and *Pld2*-KO mice displayed increased IFNγ, FasL, granzyme B and CD107a expression (Fig. 3g), whereas activated CD4⁺ T cells showed increased IFNγ⁺ and IL-17A⁺ populations with reduced FoxP3⁺ T_regs_ relative to those in WT controls (Supplementary Fig. 3g). Collectively, these findings demonstrate that PLD1/2 deficiency promotes Th1 and Th17 polarization, enhances CD8⁺ T cell cytotoxicity and alleviates T_reg_- and checkpoint-mediated suppression within the TME, thereby strengthening antitumor immune responses.

### PLD1/2-driven TAM polarization promotes tumor growth by suppressing M1 macrophage activation and T cell-mediated antitumor immunity

**Fig. 4:**
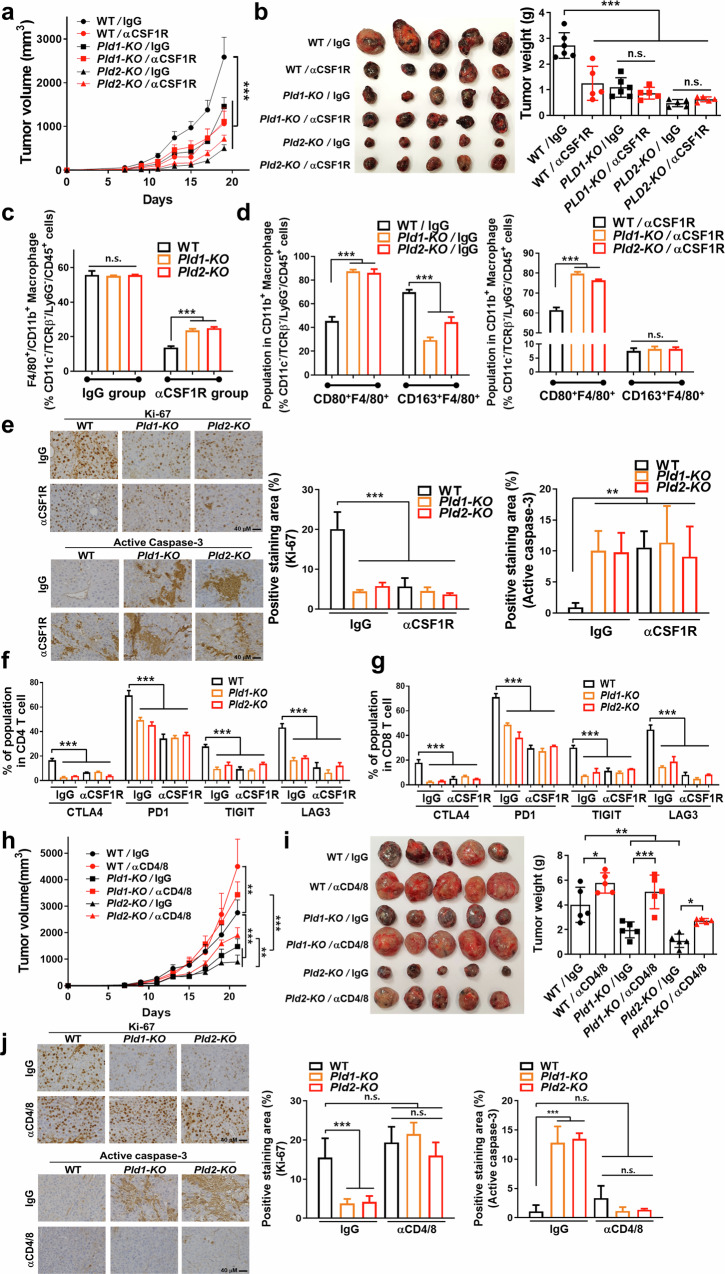
PLD1/2-driven TAM polarization promotes tumor growth by suppressing M1 macrophage activation and T cell-mediated antitumor immunity. **a**, **b** Tumor growth curves (**a**) and representative images of excised tumors with corresponding weights (**b**) from mice bearing B16F10 tumors treated with control IgG or αCSF1R antibody to deplete TAMs. Tumor volume was monitored for 19 days following tumor cell implantation. **c** Flow cytometric quantification of F4/80⁺/CD11b⁺ macrophages in tumors from IgG- or αCSF1R-treated mice. **d** Proportions of CD80⁺ (M1) and CD163⁺ (M2) macrophages among tumor-infiltrating macrophages. **e** Representative IHC images and quantification of Ki-67 and active caspase-3 expression in tumor sections from IgG- and αCSF1R-treated mice. **f**, **g** Flow cytometric analysis of the indicated immune checkpoint molecules in tumor-infiltrating CD4^+^ and CD8^+^ T cells from IgG- and αCSF1R-treated groups. **h**, **i** Tumor growth curves (**h**) and representative tumor images with corresponding weights (**i**) from tumor-bearing mice following T cell depletion using αCD4 and αCD8a antibodies. Tumor volume was measured for 22 days after tumor cell injection. **j** Representative IHC images and quantification of Ki-67 and cleaved caspase-3 in tumors from IgG- or αCD4/αCD8a-treated mice. Scale bar, 20 μm. Data are presented as mean ± s.e.m.; *P < 0.05; **P < 0.01; ***P < 0.001; n.s not significant.

We performed in vivo immune cell depletion targeting macrophages and T cells to define contributions of PLD1/2-driven TAM polarization to tumor progression and T cell-dependent immunity. WT or *Pld1*/*2*-KO B16F10 tumor-bearing mice received intraperitoneal αCSF1R every 2 days following implantation to ablate TAMs. Despite efficient TAM depletion (Supplementary Fig. 4a), *Pld1*/*2*-KO mice retained robust tumor suppression (Fig. 4a, b), indicating macrophage loss alone could not account for PLD deficiency-associated tumor control. Given enrichment of M1 macrophages in *Pld1*/*2*-KO tumors, we assessed effects of αCSF1R treatment on macrophage subsets. CSF1R blockade preferentially eliminated M2-like TAMs while preserving M1 macrophages (Fig. 4c, d and Supplementary Fig. 4b), maintaining a pro-inflammatory macrophage compartment. Consistently, IHC analyses of *Pld1*/*2*-KO tumors showed reduced Ki-67 and increased active caspase-3 staining (Fig. 4e). In WT tumors, αCSF1R treatment also reduced tumor burden, consistent with protumorigenic TAM functions^27,28^, and residual M1 macrophages contributed to partial suppression (Fig. 4d). Reduced expression of immune checkpoint molecules, including CTLA4, PD1, TIGIT and LAG3, on CD4⁺ and CD8⁺ T cells in *Pld1*/*2*-KO tumors persisted following TAM depletion (Fig. 4f, g and Supplementary Fig. 4c, d), indicating sustained T cell activation with limited exhaustion.

We depleted CD4⁺ and CD8⁺ T cells in *Pld1*/*2*-KO tumor-bearing mice using αCD4 and αCD8 antibodies to define T cell contributions to PLD deficiency-mediated tumor control (Supplementary Fig. 4e). T cell depletion abolished tumor suppression, leading to rapid regrowth (Fig. 4h, i). IHC analyses confirmed restored Ki-67 expression with reduced active caspase-3 staining in T cell-depleted tumors relative to IgG controls (Fig. 4j). Collectively, these findings demonstrate PLD1/2-driven TAM polarization promotes tumor progression by suppressing M1 macrophage activity and impairing T cell-mediated antitumor immunity, whereas PLD deficiency reprograms TAMs toward a pro-inflammatory M1 phenotype that sustains effective T cell effector function.

### PLD1/2 induce CCL19-driven M2 macrophage polarization to shape an immunosuppressive TME

**Fig. 5:**
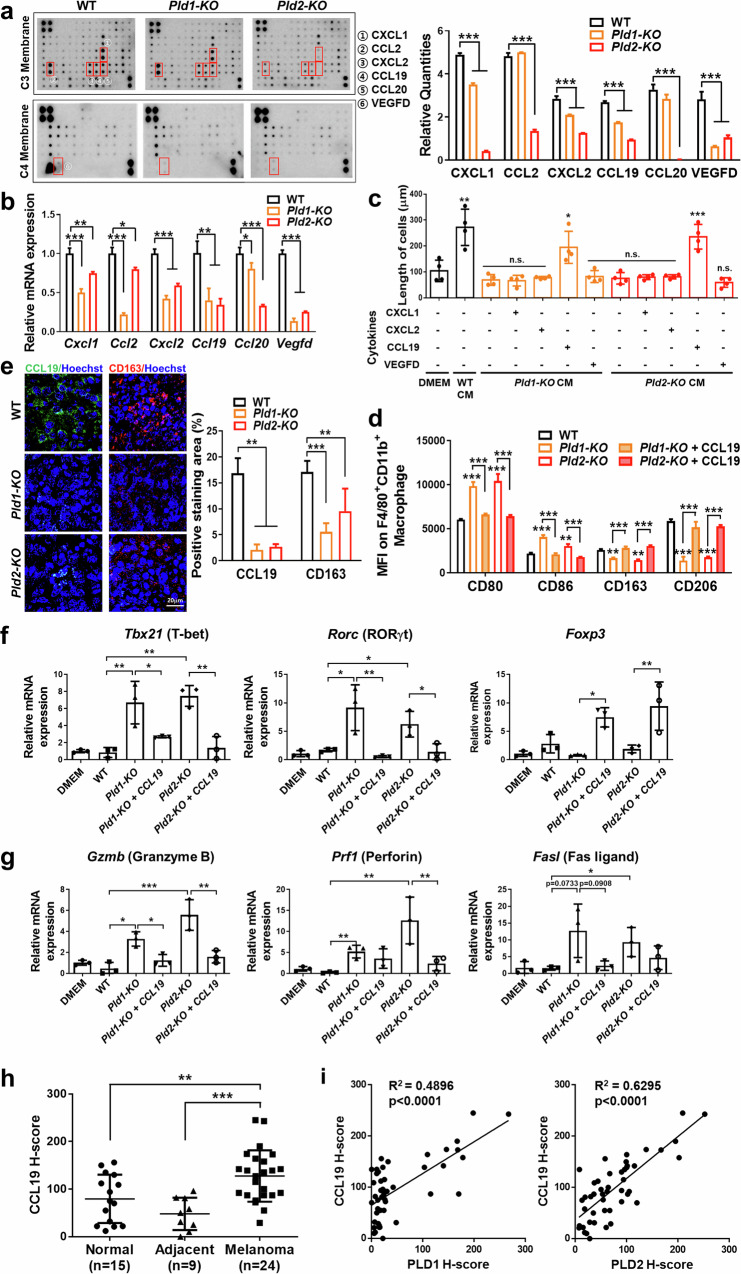
PLD1/2 induce CCL19-driven M2 macrophage polarization to shape an immunosuppressive TME. **a** Representative cytokine array images of CM from WT and *Pld1/2-KO* B16F10 cells. **b** qRT–PCR analysis of cytokine mRNA levels in the indicated cells. **c** Quantification of cell length in BMDMs treated with CM from the indicated lines. **d** Flow cytometric analysis of macrophage surface markers following CM treatment with or without recombinant CCL19. MFI, mean fluorescence intensity. **e** Representative IF images of CCL19 and CD163 in tumor tissues. **f**, **g** qRT–PCR analysis of lineage markers in CD4^+^ T cells (**f**) and cytotoxic effectors in CD8^+^ T cells (**g**) co-cultured with CM-primed BMDMs. **h**, **i** Quantification of CCL19 expression (**h**) and correlation with PLD1 and PLD2 levels (**i**) in melanoma TMA. Data are presented as mean ± s.e.m.; *P < 0.05; **P < 0.01; ***P < 0.001; n.s not significant.

Given the role of PLD1/2 in promoting immune evasion, we hypothesized that soluble factors secreted by melanoma cells mediate these effects. Cytokine array analysis of CM from WT, *Pld1*-KO and *Pld2*-KO B16F10 cells revealed reduced secretion of CXCL1, CCL2, CXCL2, CCL19, CCL20 and VEGF-D in *Pld1/2*-KO CM (Fig. 5a and Supplementary Fig. 5a), consistent with decreased mRNA expression of these cytokines (Fig. 5b). The role of CXCL1, CXCL2, CCL19 and VEGF-D were functionally validated using macrophage polarization assays. WT CM induced an elongated, spindle-shaped M2-like morphology, whereas *Pld1/2*-KO CM promoted a rounded M1-like morphology, consistent with previous reports^29,30^ (Fig. 5c and Supplementary Fig. 5b). Notably, exogenous CCL19 restored the M2-like morphology of macrophages exposed to *Pld1/2*-KO CM (Fig. 5c and Supplementary Fig. 5b). Furthermore, in TCGA melanoma datasets, CCL19 positively correlated with the M2 marker CD163 (Supplementary Fig. 5c). Flow cytometry confirmed that *Pld1/2*-KO CM induced M1 repolarization characterized by increased CD80⁺ and CD86⁺ and decreased CD163⁺ and CD206⁺ macrophages; CCL19 addition reversed these changes (Fig. 5d and Supplementary Fig. 5d). IF staining of *Pld1/2*-KO tumors revealed reduced CCL19 and CD163 expression, which correlated with decreased tumor burden (Fig. 5e). Collectively, among the cytokines reduced in *Pld1/2*-deficient cells, CCL19 was prioritized for mechanistic investigation because it showed the most consistent and reproducible reduction across PLD1/2 perturbations, robustly rescued macrophage polarization and displayed stronger associations with PLD1/2 expression and immunosuppressive TME signatures in human datasets than other downregulated candidates.

We next examined whether *Pld1/2*-deficient melanoma cells modulate T cell activation via macrophage-mediated signaling. BMDMs were primed with *Pld1/2*-KO CM with or without CCL19 and co-cultured with splenic CD4⁺ or CD8⁺ T cells in a Transwell system (Supplementary Fig. 5e). CD4⁺ T cells co-cultured with *Pld1/2*-KO CM-primed BMDMs exhibited increased *Tbx21* and *Rorc* expression and reduced *Foxp3*. CCL19 suppressed *Tbx21* and *Rorc* and increased *Foxp3*, indicating a regulatory shift (Fig. 5f). Similarly, CD8⁺ T cells co-cultured with *Pld1/2*-KO CM-primed BMDMs upregulated *Gzmb*, *Prf1* and *Fasl*, which were suppressed by CCL19 (Fig. 5g).

IHC revealed elevated CCL19 expression in human skin cancer specimens, notably in melanoma, squamous cell carcinoma, basal cell carcinoma and metastatic lesions, relative to that in adjacent normal tissues (Fig. 5h and Supplementary Fig. 6a, b). CCL19 expression positively correlated with PLD1 (*R*² = 0.4896) and PLD2 (*R*² = 0.6295) in melanoma tissues and in Gene Expression Omnibus (GEO) datasets (Fig. 5i and Supplementary Fig. 6c). These findings suggest that CCL19 could function as a PLD1/2 effector that promotes M2 macrophage polarization and establishes an immunosuppressive TME, thereby attenuating T cell-mediated antitumor immunity.

### CCL19 induces PD-L1 expression through the PI3K–Akt–NF-κB signaling axis in the TME

To evaluate the clinical relevance of this axis, we analyzed TCGA datasets across multiple cancer types, including lung adenocarcinoma, pancreatic adenocarcinoma and skin cutaneous melanoma. In nearly all gene–cancer combinations, *PD-L1* expression correlated positively with *CCL19*, *PLD1* and *PLD2*, indicating that this immunosuppressive signaling module is conserved across tumor contexts (Supplementary Fig. 7a, b).

**Fig. 6:**
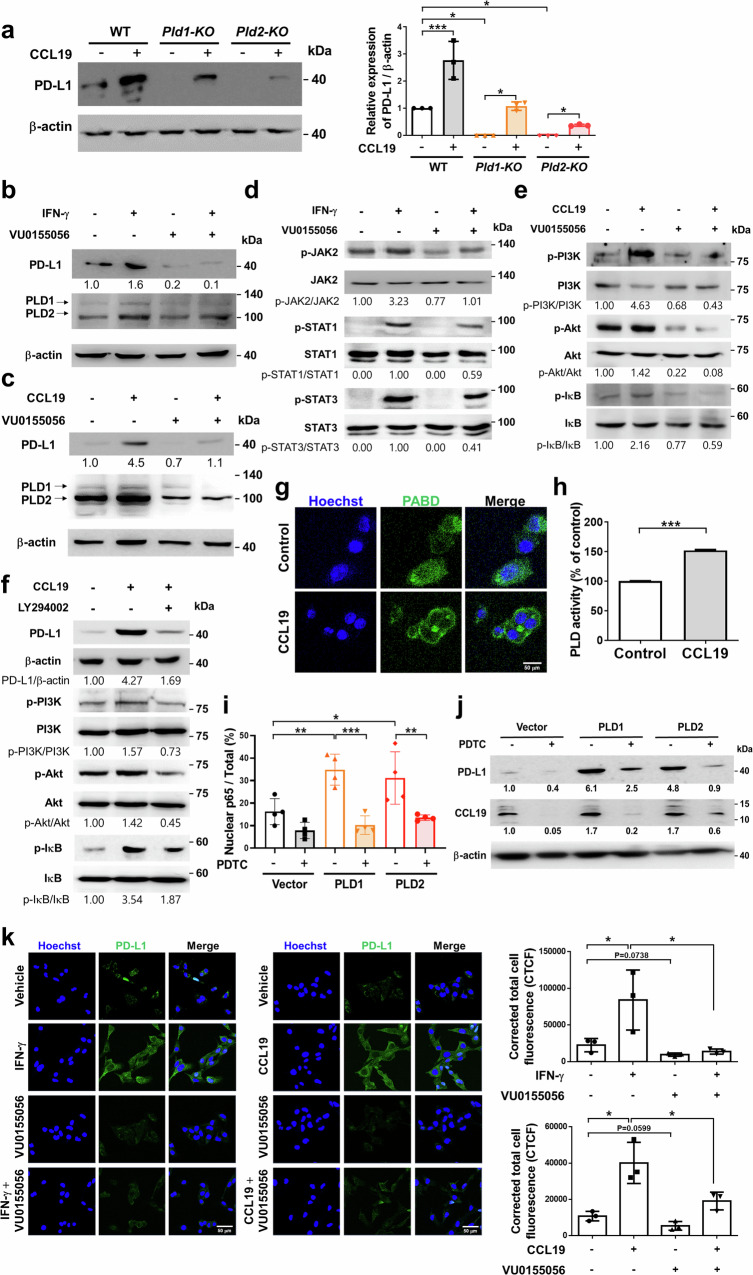
CCL19 induces PD-L1 expression through the PI3K–Akt–NF-κB signaling axis in the TME. **a** Immunoblot of PD-L1 expression in B16F10 cells treated with CCL19 (50 ng/ml) for 24 h. **b**, **c** Immunoblots of indicated proteins in B16F10 cells treated with IFN-γ (50 ng/ml) or CCL19 (50 ng/ml) with or without VU0155056 (2 μM) for 24 h. **d** Immunoblots of JAK–STAT pathway activation in serum-starved B16F10 cells treated with IFN-γ ± VU0155056 for 30 min. **e**, **f** PI3K–Akt–NF-κB pathway activation in serum-starved B16F10 cells treated with CCL19 ± VU0155056 (**e**) or ± LY294002 (**f**) for 30 min. **g** Representative frames showing PLD activation in live B16F10 cells using PABD–EGFP after 1-h CCL19 treatment. **h** Quantification of PLD activity using Amplex Red-based assay for phosphatidylcholine hydrolysis after 24-h CCL19 treatment. **i** Quantification of NF-κB p65 nuclear translocation in IF-stained B16F10 cells overexpressing PLD1 or PLD2 ± PDTC (1 μM). **j**, Immunoblot of PD-L1 and CCL19 in B16F10 cells overexpressing PLD1 or PLD2 ± PDTC (1 μM). **k** Representative IF images and quantification of PD-L1 expression following IFN-γ or CCL19 treatment ± VU0155056. Data are presented as mean ± s.e.m.; *P < 0.05; **P < 0.01; ***P < 0.001; n.s not significant.

Consistent with these patterns, PD-L1 expression was reduced in *Pld1/2*-KO melanoma cells and restored by exogenous CCL19 (Fig. 6a), demonstrating the essential role of PLD1/2-dependent CCL19 in sustaining PD-L1 expression and maintaining immunosuppression. IFN-γ reportedly induces PD-L1 via JAK–STAT signaling in melanoma and other tumor types^31,32^. Concordantly, IFN-γ increased PD-L1 expression in a time-dependent manner in B16F10 cells (Supplementary Fig. 7c), while also upregulating PLD1 and PLD2, suggesting a potential feedback mechanism. Similarly, CCL19 stimulation progressively increased PD-L1, PLD1 and PLD2 expression (Supplementary Fig. 7d), supporting a PLD1/2–CCL19–PD-L1 feedback loop potentially amplified by IFN-γ. Pharmacological inhibition of PLD1/2 with VU0155056^33^ suppressed IFN-γ- and CCL19-induced upregulation of PD-L1, PLD1 and PLD2 (Fig. 6b, c), indicating that enzymatic activity is required for PD-L1 induction under inflammatory conditions.

To elucidate the underlying pathways, we first evaluated canonical JAK–STAT activation in response to IFN-γ. IFN-γ robustly induced phosphorylation of JAK2, STAT1 and STAT3, whereas VU0155056 treatment completely blocked this activation (Fig. 6d). By contrast, CCL19 enhanced PD-L1 expression by activating the PI3K–Akt–NF-κB pathway independently of JAK–STAT (Supplementary Fig. 7e). Specifically, CCL19 induced phosphorylation of PI3K, Akt and IκB, whereas PLD inhibition substantially suppressed this cascade (Fig. 6e). Moreover, the PI3K inhibitor LY294002 abolished CCL19-induced PD-L1 expression and concomitantly blocked phosphorylation of PI3K, Akt and IκB (Fig. 6f), confirming that CCL19 induces PD-L1 expression via the PI3K–Akt–NF-κB axis.

We also observed that CCL19 induced rapid PLD activation, as detected using a GFP-based PA biosensor assay, upon short-term stimulation, and maintained elevated PLD activity, as measured via increased choline production, following long-term (24 h) treatment (Fig. 6g, h). Transient overexpression of PLD1 or PLD2 substantially increased NF-κB activation, as evidenced by nuclear translocation of p65, an effect that was abolished by the NF-κB inhibitor pyrrolidine dithiocarbamate (PDTC; Fig. 6i and Supplementary Fig. 7f). Consistently, PLD1 or PLD2 overexpression significantly increased PD-L1 and CCL19 levels (Fig. 6j) and *Ccl19* mRNA expression (Supplementary Fig. 7g); these effects were effectively suppressed by PDTC treatment. These findings demonstrate that PLD1/2 activate NF-κB signaling to drive a pro-inflammatory transcriptional program that induces CCL19 and upregulates PD-L1. Finally, IF staining confirmed that IFN-γ- or CCL19-mediated PD-L1 induction was completely abolished by VU0155056-induced PLD inhibition (Fig. 6k), further establishing that PLD enzymatic activity is indispensable for PD-L1 upregulation. Collectively, these findings suggest that PLD1/2-driven CCL19 production promotes PD-L1 expression by activating the PI3K–Akt–NF-κB signaling axis, thereby establishing a feed-forward loop that reinforces immunosuppression within the TME.

### Dual inhibition of PLD1/2 suppresses tumor growth by reprogramming the immune microenvironment

**Fig. 7:**
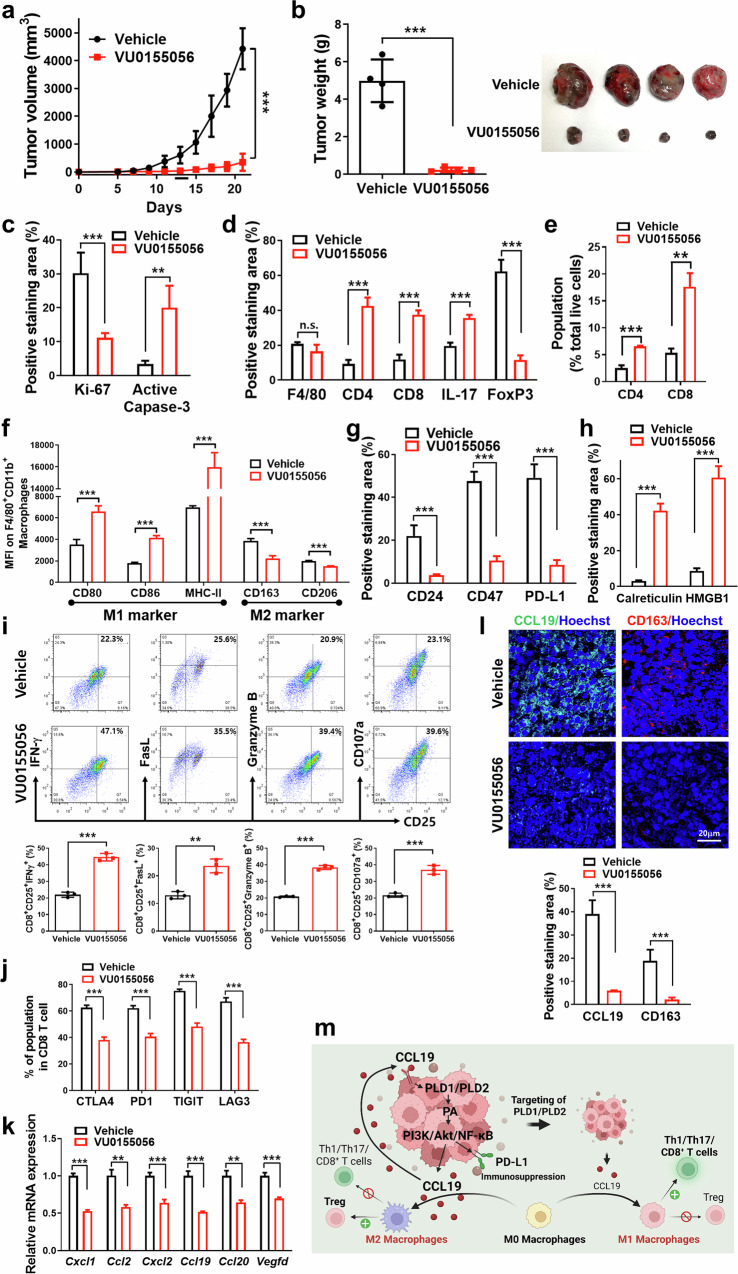
Dual inhibition of PLD1/2 suppresses tumor growth by reprogramming the immune microenvironment. C57BL/6 mice bearing B16F10 tumors were treated with VU0155056 (10 mg/kg) to evaluate therapeutic efficacy. **a**, **b** Tumor growth curves over 21 days (**a**) and representative tumor images with corresponding weights (**b**) at endpoint. **c**–**f** Tumors were collected for IHC and flow cytometric analyses. The image shows quantification of Ki-67 and active caspase-3 expression in tumor tissues (**c**), quantification of indicated markers in IHC-stained sections (**d**), flow cytometric quantification of total CD4^+^ or CD8^+^ T cells among total live tumor-infiltrating lymphocytes (**e**) and flow cytometric analysis of the indicated markers in tumors from vehicle- and VU0155056-treated mice (**f**). **g**, **h** Quantification of phagocytosis-related checkpoint molecule expression, showing 'don't eat me' signals (CD24, CD47 and PD-L1) in **g** and 'eat me' signals (calreticulin and HMGB1) in **h**. **i** Flow cytometric analysis of cytotoxic CD8^+^/CD25^+^ T cells expressing IFNγ, FasL, granzyme B and CD107a. **j** Flow cytometric analysis of immune checkpoint receptors on tumor-infiltrating CD8⁺/CD25⁺ T cells. **k** qRT–PCR analysis of cytokine expression in B16F10 cells treated with VU0155056 (5 μM, 24 h). **l** Representative IF staining of CCL19 and CD163 in tumor tissues from vehicle- and VU0155056-treated mice. **m** Schematic illustrating PLD1/2-dependent CCL19 secretion, M2 macrophage polarization and PD-L1 induction, resulting in the establishment of an immunosuppressive TME. Data are presented as mean ± s.e.m.; **P < 0.01; ***P < 0.001. Schematic in **m** created in BioRender.Min, D. S. (2026) https://BioRender.com/ddx8qce.

The dual PLD1/2 inhibitor VU0155056, a potent small-molecule inhibitor^33^, was used to assess whether pharmacological inhibition recapitulates the immunomodulatory effects observed in genetic models. Systemic administration of VU0155056 substantially suppressed tumor growth in B16F10 melanoma-bearing mice (Fig. 7a,b) and significantly reduced spleen size and weight compared with those in vehicle-treated controls (Supplementary Fig. 8a). IHC analyses revealed decreased Ki-67 staining and increased caspase-3 activation, indicating reduced proliferation and enhanced apoptosis following treatment (Fig. 7c and Supplementary Fig. 8b).

Although total F4/80⁺ macrophage abundance remained unchanged, VU0155056 treatment significantly increased intratumoral CD4⁺ and CD8⁺ T cell infiltration, recapitulating immune phenotypes in Pld1- and Pld2-deficient tumors (Fig. 7d and Supplementary Fig. 8c). IL-17⁺ cells were significantly elevated, whereas FoxP3⁺ T_regs_ were reduced, indicating a shift toward a pro-inflammatory, antitumor T cell milieu (Fig. 7d and Supplementary Fig. 8c). These findings were corroborated by flow cytometry, which confirmed enhanced effector T cell infiltration (Fig. 7e and Supplementary Fig. 8d).

Despite no change in macrophage numbers, flow cytometry revealed a pronounced increase in the M1/M2 ratio, reflecting a polarization shift toward M1-like macrophages following VU0155056 treatment (Fig. 7f and Supplementary Fig. 8e). Concurrently, expression of ‘don’t eat me’ signals (CD24, CD47 and PD-L1) was significantly reduced (Fig. 7g and Supplementary Fig. 8f), whereas ‘eat me’ signals (calreticulin and HMGB1) were upregulated (Fig. 7h and Supplementary Fig. 8g), indicating enhanced immunogenic phagocytosis within the TME. Functional profiling of tumor-infiltrating T cells demonstrated that VU0155056 significantly increased the proportions of IFNγ⁺, FasL⁺, granzyme B⁺ and CD107a⁺ CD8⁺ T cells, indicative of heightened cytotoxic activity (Fig. 7i). Similarly, CD4^+^ T cells exhibited significantly increased proportions of IFN-γ^+^ and IL-17a^+^ cells, along with a reduction in FoxP3^+^ T_regs_, further supporting a shift toward a pro-inflammatory effector phenotype within the TME (Supplementary Fig. 8h). Immune checkpoint receptors (PD1, CTLA4, TIGIT and LAG3) were downregulated in both CD8⁺ and CD4⁺ T cells (Fig. 7j and Supplementary Fig. 8i,j), indicating reduced T cell exhaustion and enhanced effector function.

Consistent with immune phenotypes in *Pld1/2*-deficient tumors, VU0155056 significantly reduced six previously identified PLD-dependent cytokines, with CCL19 exhibiting the most pronounced decrease (Fig. 7k,l). Given that CCL19 promotes M2 macrophage polarization and induces PD-L1 expression via the PI3K–Akt–NF-κB axis, its suppression probably contributes to the establishment of an antitumor immune microenvironment (Fig. 7m). These findings demonstrate that pharmacological inhibition with the dual PLD1/2 inhibitor VU0155056 effectively suppresses melanoma growth by reprogramming the immune landscape, enhancing T cell cytotoxicity, promoting M1 macrophage polarization and attenuating key immunosuppressive mediators such as CCL19 and PD-L1. These results support dual PLD1/2 inhibition as a promising strategy to potentiate cancer immunotherapy by fostering a more immunostimulatory TME.

## Discussion

PLD has long been recognized for roles in regulating membrane dynamics, vesicle trafficking and mitogenic signaling through PA generation^18,34^. Aberrant activation of PLD1 and PLD2 has been widely implicated in tumor growth, invasion and therapy resistance across multiple malignancies^18,25^. However, previous work has largely emphasized tumor-intrinsic functions of PLD, including proliferation, survival and metabolic adaptation, leaving its role in shaping the tumor immune microenvironment insufficiently defined. Here, we identified tumor-intrinsic PLD1 and PLD2 as central regulators of immunosuppression in melanoma. We demonstrated that PLD1/2 activity coordinates macrophage polarization, cytokine secretion and immune checkpoint regulation, thereby remodeling the immune landscape of the TME. Mechanistically, PLD1/2 drives CCL19 production, establishing a PLD–CCL19–PD-L1 signaling axis that links phospholipid metabolism with immune evasion. Although PLD signaling potentiates oncogenic pathways such as mTOR, RAS–MAPK, PI3K–Akt and Wnt–β-catenin^35–37^, these findings extend established functions by revealing coordinated immunoregulatory activity within the TME. Notably, PLD1/2-mediated CCL19 production enables PLD to exert control over stromal and tumor-intrinsic immune compartments, establishing a self-reinforcing immunosuppressive circuit that sustains immune evasion.

Previous studies suggested participation of PLD in tumor-associated inflammation, although direct evidence defining immunoregulatory functions within the TME remains limited. PLD1 inhibition has been linked to immunogenic cell death and enhanced macrophage-mediated phagocytosis in colorectal cancer models^19^, and other reports have associated PLD signaling with cytokine production and immune cell recruitment in diverse tumor contexts^38–40^. In addition, PLD2 has been implicated in regulating CD8⁺ T cell proliferation and antitumor activity in vivo, supporting broader roles in immune modulation^20^. However, much of this work emphasized inflammatory or tumor-promoting outcomes rather than mechanisms governing immune suppression. Importantly, these studies largely described PLD as a general modulator of inflammatory cytokine production or immune cell recruitment and did not delineate a tumor-intrinsic signaling circuit linking lipid metabolism to coordinated macrophage reprogramming and immune checkpoint regulation within the TME. Our findings integrate and extend previous observations by demonstrating that PLD1 and PLD2 amplify inflammatory signaling while orchestrating immune suppression through coordinated regulation of cytokine signaling and immune checkpoint expression. In contrast to previously reported inflammatory roles, our study identifies a PLD1/2–CCL19–PD-L1 feed-forward axis linking tumor lipid signaling to macrophage polarization and checkpoint regulation. To our knowledge, this study provides the first mechanistic evidence linking tumor lipid signaling to coordinated macrophage reprogramming and immune checkpoint regulation in the TME.

Identification of CCL19 as a downstream effector of PLD activity adds a critical layer to this regulatory network. Although CCL19 is classically associated with the CCR7-dependent trafficking of T cells and dendritic cells^41–43^, its role in cancer appears to be highly context dependent. Although CCL19 has been reported to exert antitumor effects through enhanced immune recruitment in certain settings^41^, accumulating evidence also implicates CCL19 in tumor progression and immune evasion^42,44^.

Our findings support a predominantly immunosuppressive function in melanoma, whereby CCL19 promotes M2 macrophage polarization and induces PD-L1 expression. These data suggest that CCL19–CCR7 signaling may function as a molecular rheostat balancing immune activation and suppression within the TME. Future studies will be required to delineate effects of CCL19 on dendritic cells and regulatory T cells, both expressing CCR7, to define the full immunologic spectrum within this context.

From a therapeutic perspective, PLD1/2 inhibition may represent an immunomodulatory strategy to overcome tumor immune evasion. Given the association between elevated PD-L1 expression, poor prognosis and resistance to ICB^45,46^, targeting the PLD–CCL19–PD-L1 axis could enhance the efficacy of existing immunotherapies.

Despite these promising findings, this study had several limitations that warrant consideration. First, the relative contributions of PLD1 and PLD2 remain to be fully defined. Although both isoforms appear to be functionally redundant in driving CCL19 secretion and immune suppression, isoform-specific KO or rescue studies are required to delineate potential differences in their downstream signaling mechanisms. Second, our analyses were performed primarily in melanoma models, and whether the PLD–CCL19–PD-L1 axis operates similarly across other tumor types and immune contexts remains unclear. Third, although we identified PI3K–Akt–NF-κB signaling as the principal pathway mediating CCL19-induced PD-L1 expression, additional signaling pathways, including MAPK cascades, may also contribute to PLD-driven immune regulation. Further mechanistic and translational studies are required to address these questions.

In conclusion, we identified a previously unrecognized immunoregulatory role for PLD1 and PLD2 in remodeling the TME through chemokine-driven macrophage polarization and PD-L1 induction. PLD inhibition represents a therapeutic strategy targeting tumor-intrinsic oncogenic signaling and extrinsic immune suppression. Further evaluation of PLD inhibitors combined with ICB may reveal synergistic antitumor effects and facilitate the development of next-generation immunomodulatory therapies.

## Supplementary information

Supplementary Information

## Data Availability

TCGA datasets analyzed in this study were accessed through cBioPortal and TIMER2.0. All other raw data generated in this study are available upon request from the corresponding author.
