# Supplementary material for: PLD1 and PLD2 promote an immunosuppressive tumor microenvironment via CCL19-dependent macrophage polarization and PD-L1 induction
Source: Exp Mol Med. 2026 Jun 4;58(6):1870–84. doi: 10.1038/s12276-026-01742-y (PMC13324033; doi:10.1038/s12276-026-01742-y)
Supplement: Supplementary file 1 — Supplementary Information [file 12276_2026_1742_MOESM1_ESM.pdf]

PLD1 and PLD2 promote an immunosuppressive tumor microenvironment via CCL19-dependent macrophage polarization and PD-L1 induction

This file includes:

Supplementary Fig. 1 to 8

Supplementary Table 1 to 4

Supplementary Fig. 1. Expanded evaluation of PLD1/2 expression in human skin cancers and functional deficits associated with *Pld1/2* knockout in melanoma cells.

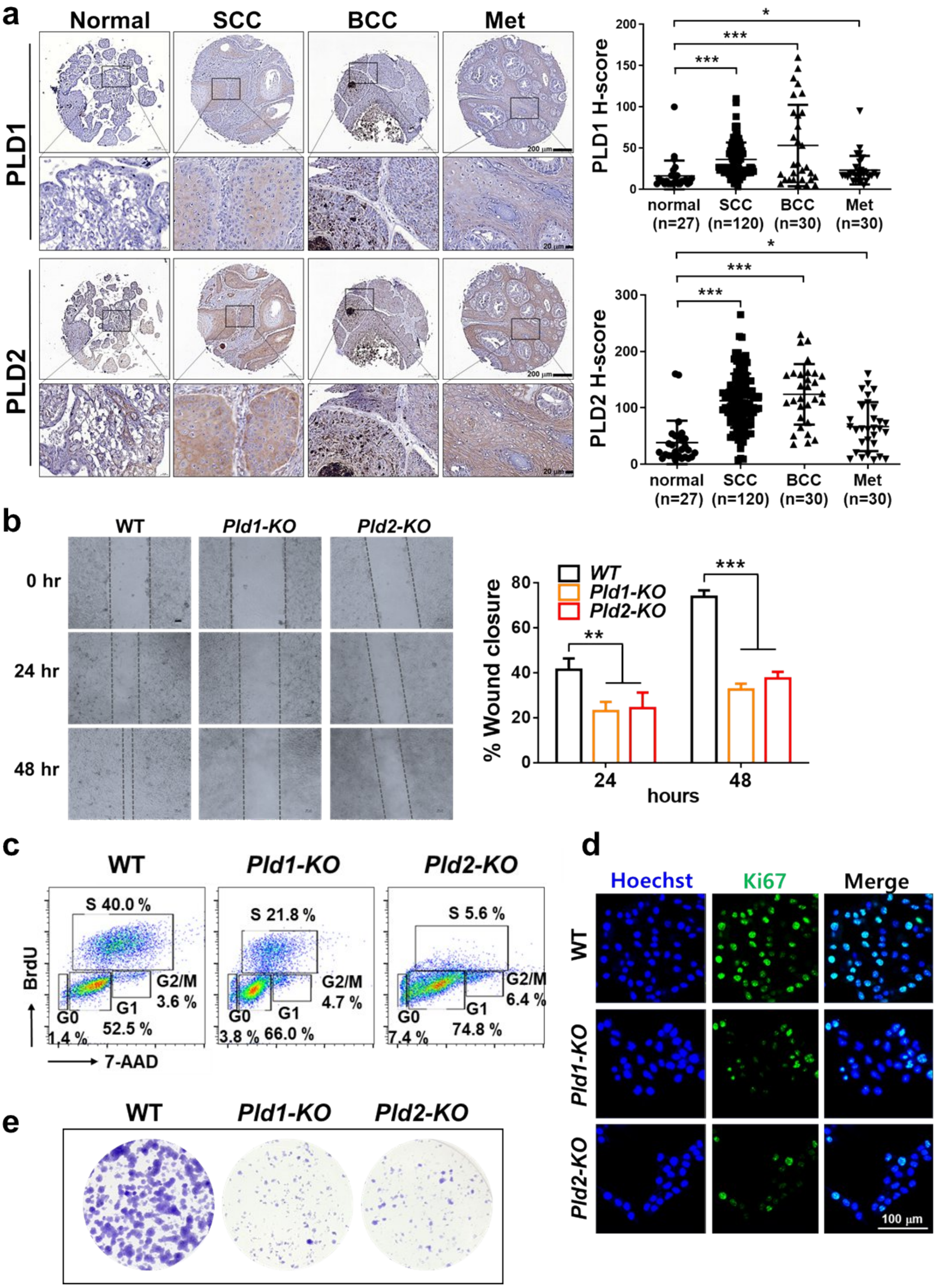

a Representative IHC images and quantification of PLD1 and PLD2 in normal skin, SCC, BCC, and Met tissue microarrays. **b** Wound-healing assays performed in WT, *Pld1-KO*, and *Pld2-KO* B16F10 cells at the indicated time points, with quantification of wound closure. **c** Cell-cycle profiles of WT and *Pld1/2-KO* B16F10 cells determined by BrdU/7-AAD flow cytometry, showing the percentage of cells in G0, G1, S, and G2/M phases. **d** Representative IF images of Ki-67 staining in WT and *Pld1/2-KO* cells, with Hoechst 33342 counterstaining. **e** Colony-formation assays showing representative crystal-violet-stained colonies from WT and *Pld1/2-KO* cells. Data are presented as mean ± SEM. * $p < 0.05$; ** $p < 0.01$; *** $p < 0.001$.

Supplementary Fig. 2. Extended characterization of spleen phenotypes, myeloid populations, and macrophage functional markers in WT and *Pld1/2*-deficient tumors

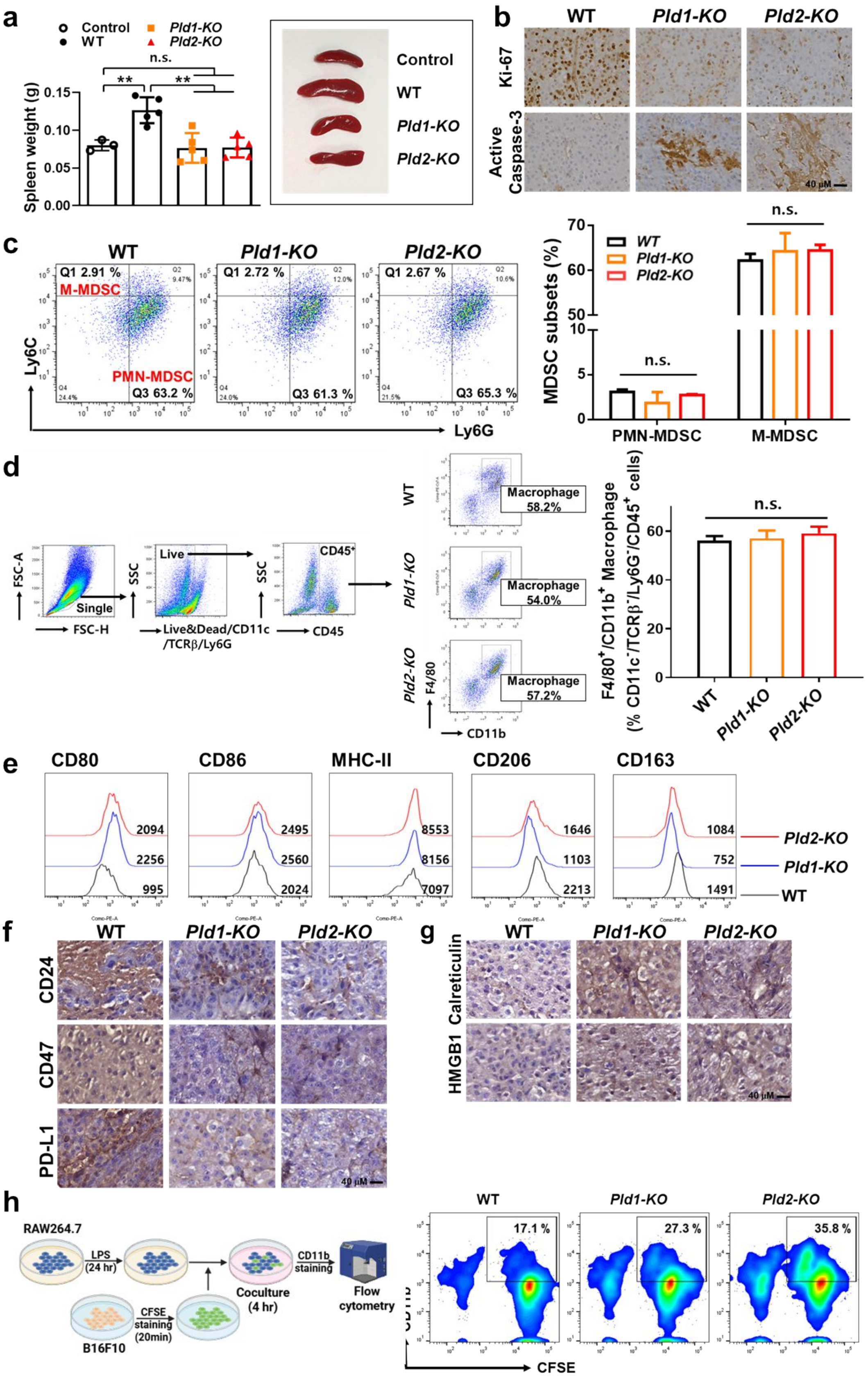

a Representative images of spleen from WT and *Pld1/2-KO* tumor-bearing mice, with corresponding spleen-weight quantification. **b** Representative IHC images of Ki-67 and active caspase-3 in tumor sections. **c** Flow-cytometric analysis of tumor-infiltrating MDSC subsets, showing the percentages of PMN-MDSCs and M-MDSCs in WT and *Pld1/2-KO* tumors, along with quantitative summary data. **d** Gating strategy for identifying tumor-infiltrating macrophages and flow-cytometric quantification of $F4/80^+/CD11b^+$ macrophages in WT and *Pld1/2-KO* tumors. **e** Flow-cytometric analysis of macrophage-associated surface markers within the $F4/80^+/CD11b^+$ population, including activation and polarization markers. **f-g** Representative images of IHC images of “don’t eat me” and “eat me” signals in tumors. **h** Schematic depiction of the LPS-activated RAW264.7 phagocytosis assay and flow-cytometric measurement of phagocytosis of WT and *Pld1/2-KO* B16F10 cells. Data are presented as mean ± SEM. *$p < 0.05$; **$p < 0.01$; ***$p < 0.001$.

Supplementary Fig. 3. Extended flow-cytometric analyses of CD4^{+} and CD8^{+} T-cell functional states in WT and *Pld1/2*-deficient tumors.

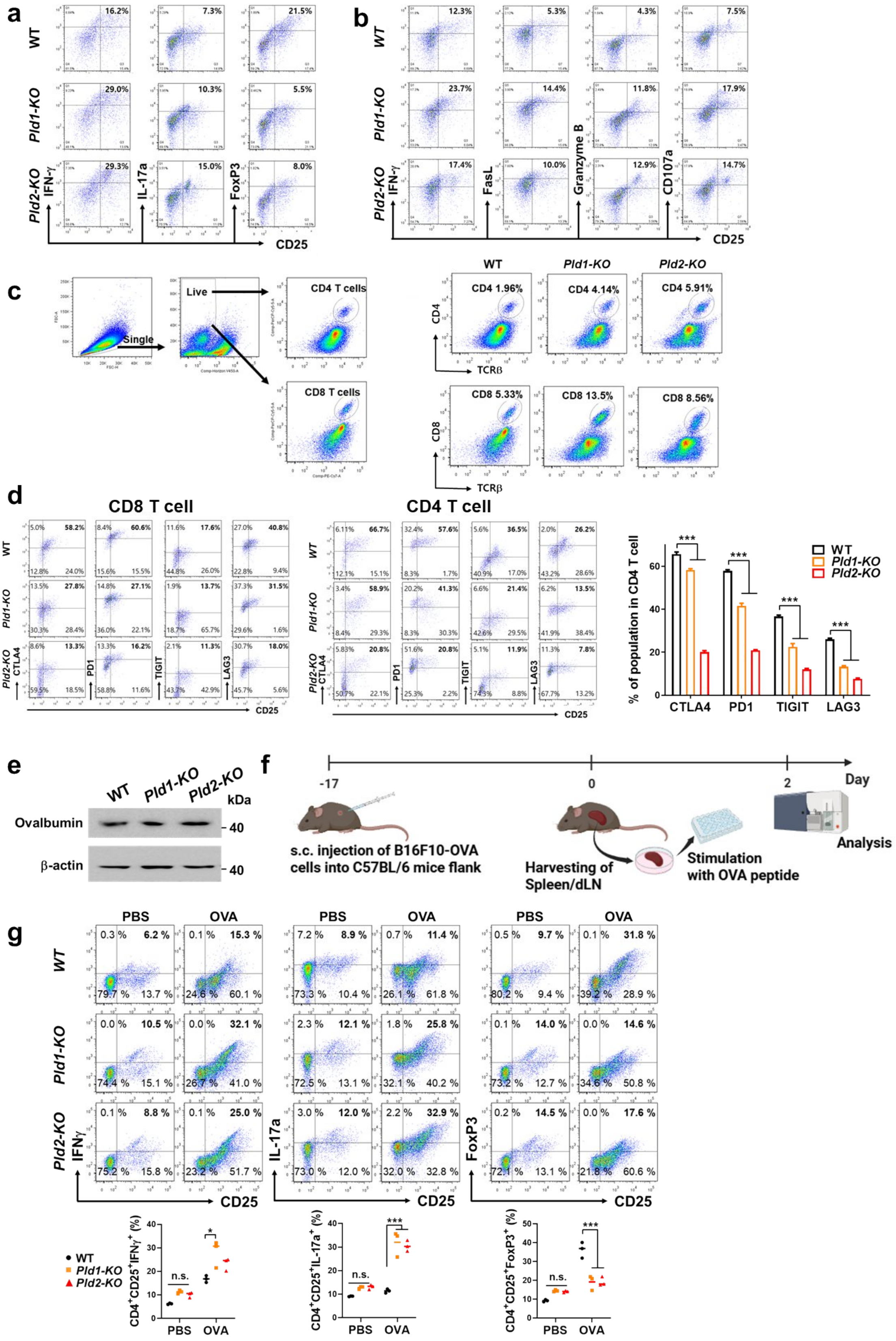

a Flow-cytometric analysis of IFNγ$^+$, IL-17a$^+$, and FoxP3$^+$ populations within CD4$^+$/CD25$^+$ tumor-infiltrating T cells from WT and *Pld1/2-KO* tumors. **b** Flow-cytometric analysis of cytotoxic effector markers in CD8$^+$/CD25$^+$ T cells, including IFNγ, FasL, granzyme B, and CD107a. **c** Gating strategy for identifying CD4$^+$ and CD8$^+$ tumor-infiltrating lymphocytes. **d** Flow-cytometric analysis of immune-checkpoint molecules expressed on CD8$^+$ and CD4$^+$ T cells. **e** Western blot analysis confirming OVA expression in WT and *Pld1/2-KO* B16F10 cells. **f** Schematic representation of the *ex vivo* OVA-specific T-cell restimulation assay using splenic T cells isolated from syngeneic tumor-bearing mice. **g** Ex vivo restimulation of splenic T cells from tumor-bearing mice with OVA peptide for 2 d, followed by flow cytometric assessment of CD4$^+$/CD25$^+$ T cells. Data are presented as mean ± SEM. *$p < 0.05$; ***$p < 0.001$.

Supplementary Fig. 4. Flow-cytometric characterization of macrophage and T-cell populations in immune-cell depletion experiments.

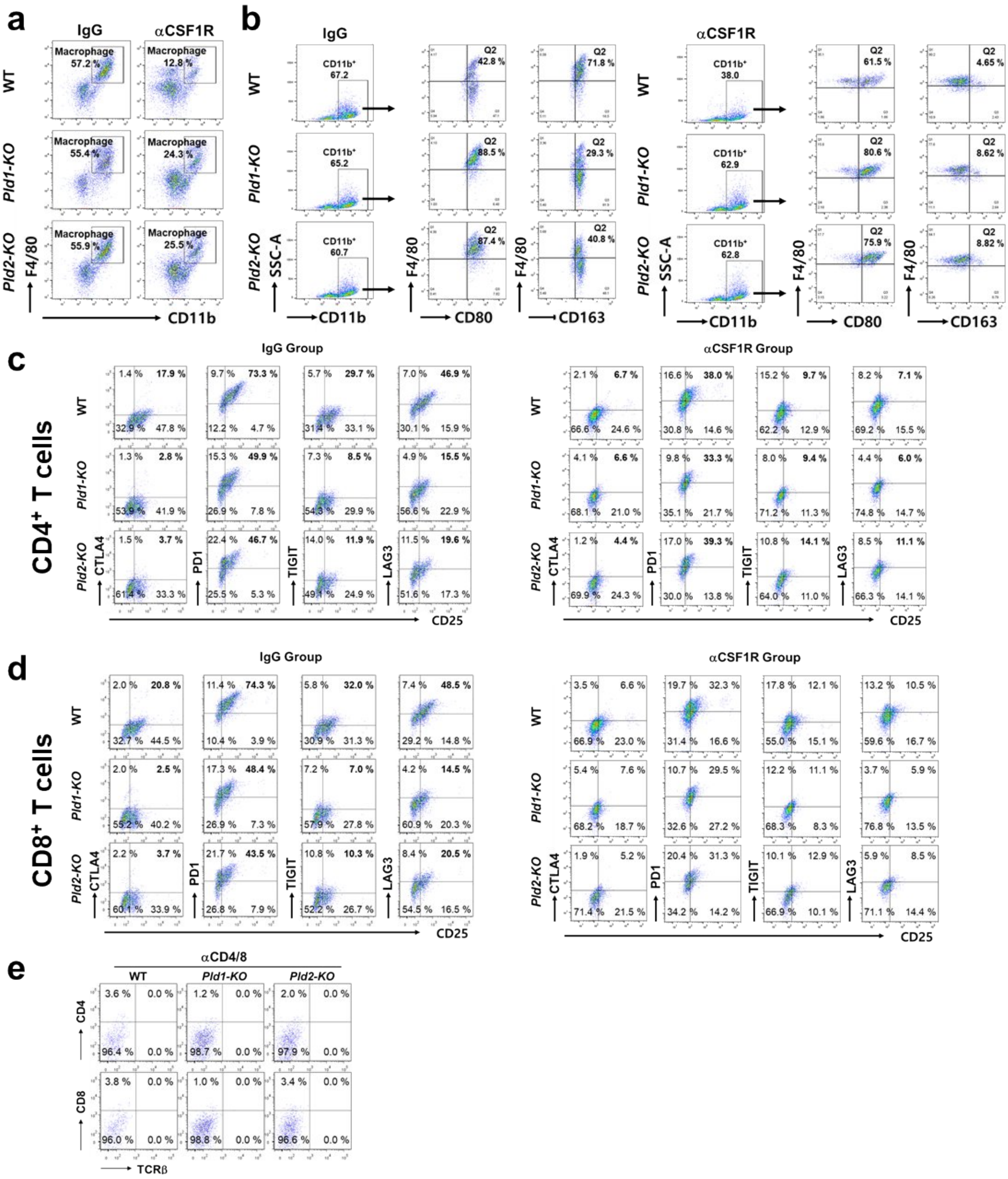

a Flow-cytometry of F4/80⁺/CD11b⁺ tumor-infiltrating macrophages from IgG- and αCSF1R-treated groups. **b** Flow-cytometry of CD80⁺ (M1) and CD163⁺ (M2) macrophages within the tumor-infiltrating macrophages population across treatment groups. **c-d** Flow-cytometric analysis of immune-checkpoint molecules expressed on tumor-infiltrating T cells from the indicated treated groups. **e** Flow-cytometric confirmation of T-cell depletion in tumors from αCD4/8 antibody–treated mice.

Supplementary Fig. 5. Additional experimental details supporting PLD1/2–CCL19–mediated macrophage and T-cell assays.

a

C3 Membrane

	A	B	C	D	E	F	G	H	I	J	K	L	M	N
1	POS	POS	NEG	NEG	BLANK	Axl	BCL	CD30L	CD30	CD40	CRG-2	CTACK	CXCL16	Eotaxin 1
2	POS	POS	NEG	NEG	BLANK	Axl	BCL	CD30L	CD30	CD40	CRG-2	CTACK	CXCL16	Eotaxin 1
3	Eotaxin 2	Fas Ligand	CX3CL1	GCSF	GM-CSF	IFN gamma	IGFBP 3	IGFBP 5	IGFBP 6	IL-1 alpha	IL-1 beta	IL-2	IL-3	IL-3 Rb
4	Eotaxin 2	Fas Ligand	CX3CL1	GCSF	GM-CSF	IFN gamma	IGFBP 3	IGFBP 5	IGFBP 6	IL-1 alpha	IL-1 beta	IL-2	IL-3	IL-3 Rb
5	IL-4	IL-5	IL-6	IL-9	IL-10	IL12 p40/70	IL12 P70	IL-13	IL-17	KC	Leptin R	Leptin	LIX	L Selectin
6	IL-4	IL-5	IL-6	IL-9	IL-10	IL12 p40/70	IL12 P70	IL-13	IL-17	KC	Leptin R	Leptin	LIX	L Selectin
7	Ltn/XCL1	MCP-1	MCP-5	M-CSF	MIG	MIP-1 alpha	MIP-1 gamma	MIP-2	MIP-3 beta	MIP-3 alpha	PF-4	P Selectin	RANTES	SCF
8	Ltn/XCL1	MCP-1	MCP-5	M-CSF	MIG	MIP-1 alpha	MIP-1 gamma	MIP-2	MIP-3 beta	MIP-3 alpha	PF-4	P Selectin	RANTES	SCF
9	SDF-1 alpha	TARC	TCA-3	TECK	TIMP-1	TNF alpha	sTNFRI	sTNFRII	TPO	VCAM-1	VEGF-A	BLANK	BLANK	POS
10	SDF-1 alpha	TARC	TCA-3	TECK	TIMP-1	TNF alpha	sTNFRI	sTNFRII	TPO	VCAM-1	VEGF-A	BLANK	BLANK	POS

C4 Membrane

	A	B	C	D	E	F	G	H	I	J	K	L
1	POS	POS	NEG	NEG	BLANK	bFGF	CD26	Dtk	E Selectin	Ec Gamma RIIB	Fit-3 Ligand	GITR
2	POS	POS	NEG	NEG	BLANK	bFGF	CD26	Dtk	E Selectin	Ec Gamma RIIB	Fit-3 Ligand	GITR
3	HGF R	ICAM-1	IGFBP 2	IGF-1	IGF-2	IL-15	IL-17B R	IL-7	I-TAC	Lungkine	MDC	MMP-2
4	HGF R	ICAM-1	IGFBP 2	IGF-1	IGF-2	IL-15	IL-17B R	IL-7	I-TAC	Lungkine	MDC	MMP-2
5	MMP-3	OPN	OPG	Pro MMP-9	Resistin	Shh-N	TCK-1	TIMP-2	TRANCE	TROY	TSLP	VEGF R1
6	MMP-3	OPN	OPG	Pro MMP-9	Resistin	Shh-N	TCK-1	TIMP-2	TRANCE	TROY	TSLP	VEGF R1
7	VEGF R2	VEGF R3	VEGF D	BLANK	BLANK	BLANK	BLANK	BLANK	BLANK	BLANK	BLANK	POS
8	VEGF R2	VEGF R3	VEGF D	BLANK	BLANK	BLANK	BLANK	BLANK	BLANK	BLANK	BLANK	POS

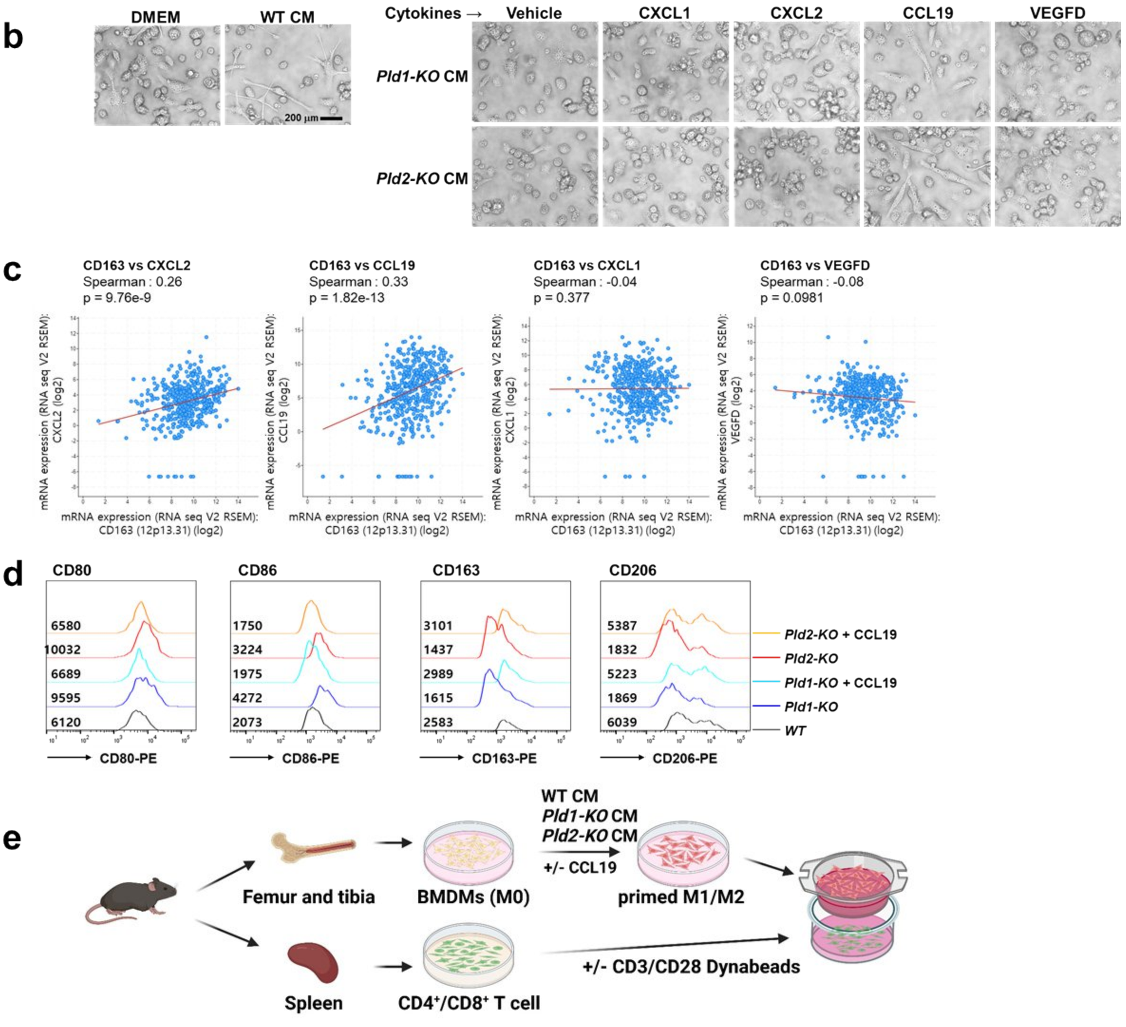

a Cytokine array layout showing the positions and identities of cytokines printed on the C3 and C4 membranes used for CM profiling. **b** Representative morphology images of BMDMs treated with CM from WT and *Pld1/2-KO* B16F10 cells with the indicated recombinant cytokines. **c** Analysis of TCGA melanoma datasets assessing the association between cytokines expression and the macrophage marker CD163. **d** Flow-cytometry analysis of macrophage surface markers in BMDMs treated with CM from WT or *Pld1/2-KO* B16F10 cells, with or without recombinant CCL19. **e** Schematic diagram of the experimental workflow in which BMDMs were primed with CM from WT or *Pld1/2-KO* cells in the presence or absence of CCL19 and subsequently co-cultured with splenic $CD4^+$ or $CD8^+$ T cells using a transwell system.

Supplementary Fig. 6. CCL19 expression and association with PLD1/2 in human skin cancer specimens.

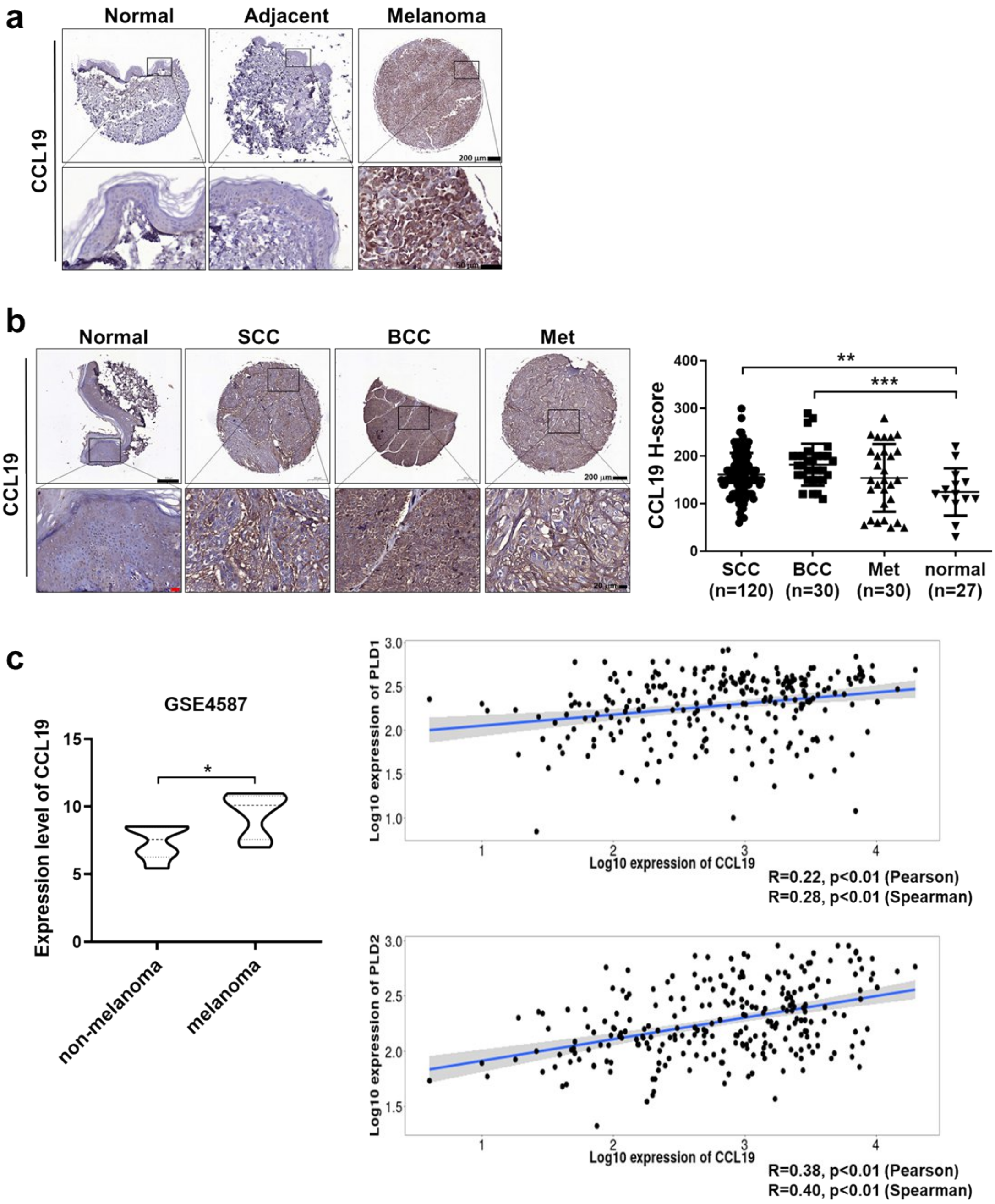

a Representative IHC images showing CCL19 expression in melanoma TMA. **b** Representati ve IHC images showing CCL19 expression in normal skin, SCC, BCC, and Met in TMA, wit h corresponding quantification of IHC scores. **c** Analysis of CCL19 expression in melanoma versus non-melanoma tissues using GEO datasets, followed by correlation analyses between CCL19 and PLD1 or PLD2 expression in melanoma tissues. Data are presented as mean ± SEM. $*p < 0.05$; $**p < 0.01$; $***p < 0.001$.

Supplementary Fig. 7. Analyses of CCL19–PLD1/2–PD-L1 signaling across cancer datasets and in melanoma cells.

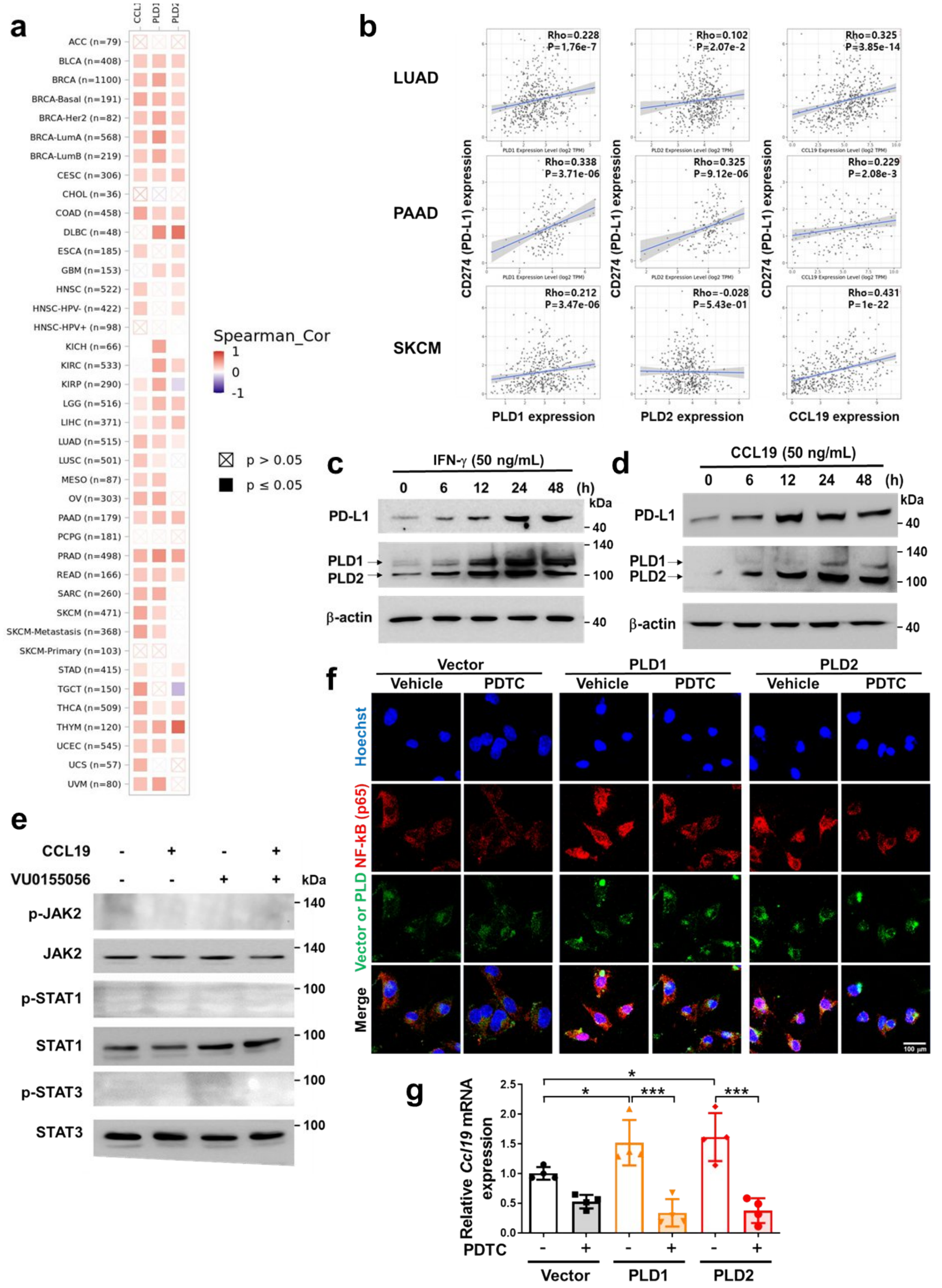

a Correlation analyses of PD-L1 with CCL19, PLD1, or PLD2 across multiple cancer types using the TIMER2.0 database. **b** Graphical representation of PD-L1 correlations with CCL19, PLD1, and PLD2 specifically in LUAD, PAAD, and SKCM. **c** Western blot analysis of PD-L1, PLD1, and PLD2 expression in B16F10 cells treated with IFN-γ (50 ng/mL) for the indicated time points. **d** Western blot analysis of PD-L1, PLD1, and PLD2 expression in B16F10 cells stimulated with CCL19 (50 ng/mL) for the indicated time points. **e** Western blot analysis of JAK2, STAT1, and STAT3 phosphorylation in B16F10 cells treated with CCL19 ± VU0155056 (2 μM). **f-g** Representative IF images (**f**) showing nuclear translocation of NF-κB in cells overexpressing GFP-tagged PLD1 or PLD2 following CCL19 stimulation, along with corresponding *Ccl19* mRNA expression (**g**).

Supplementary Fig. 8. Additional analyses of immune remodeling following pharmacologic PLD1/2 inhibition in melanoma.

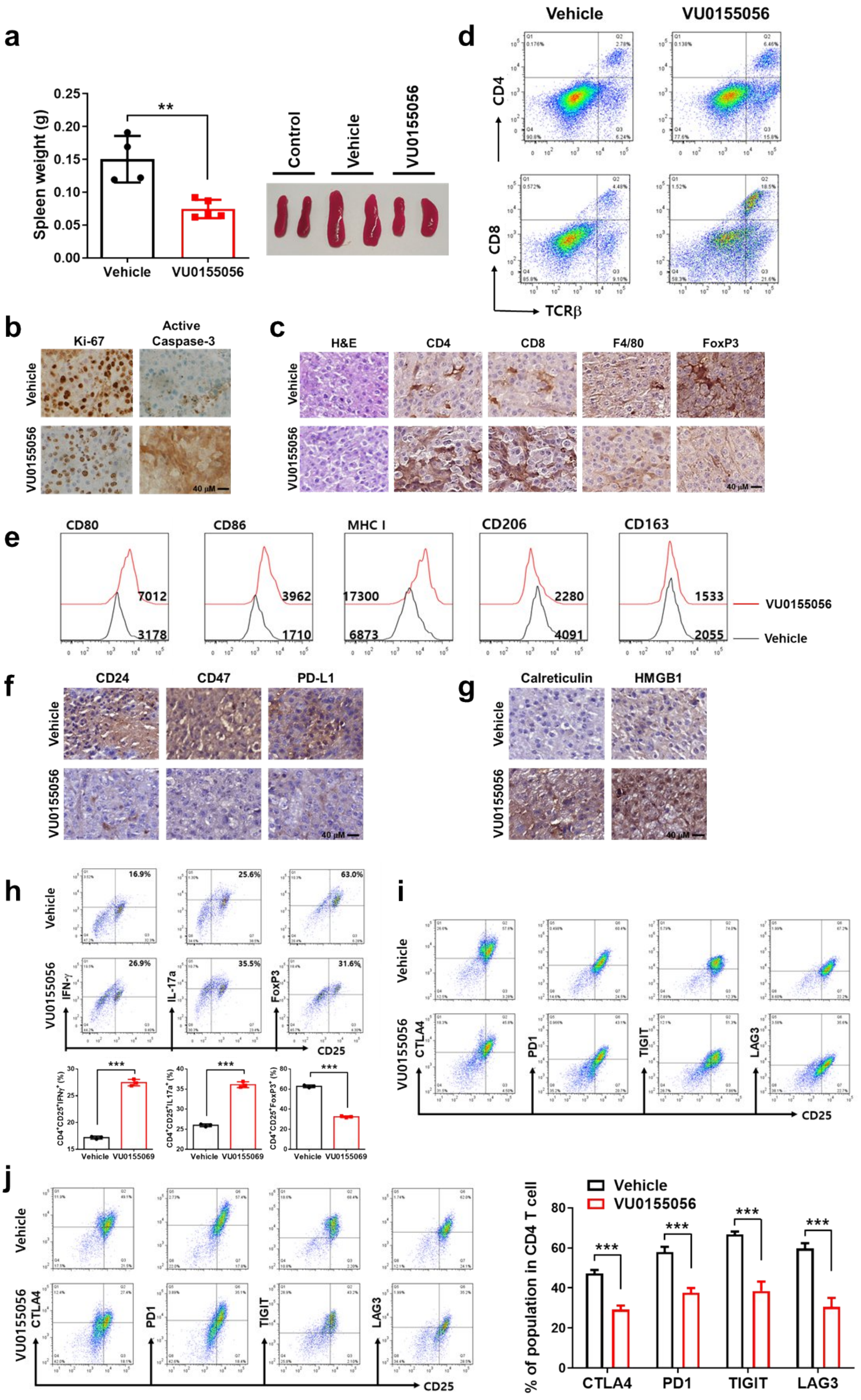

a Representative images and quantification of spleen weight from tumor-bearing mice treated with vehicle or VU0155056. **b** Representative IHC images of Ki-67 and active caspase-3 in tumor sections collected from vehicle- or VU0155056-treated mice. **c** Representative H&E and IHC images of F4/80, CD4, CD8, IL-17, and FoxP3 in tumor tissues. **d** Flow-cytometry analysis of tumor-infiltrating $CD4^+$ and $CD8^+$ T cells from vehicle- and VU0155056-treated groups. **e** Flow-cytometry analysis of macrophage markers in tumors. **f-g** Representative IHC images of "don't eat me" signals (CD24, CD47, and PD-L1) and "eat me" signals (calreticulin and HMGB1) in tumors. **h** Flow-cytometry analysis of IFN-γ^+, IL-17a$^+$, and FoxP3$^+$ populations within tumor-infiltrating $CD4^+$ T cells. **i** Flow-cytometry analysis of immune checkpoint molecules on tumor-infiltrating $CD8^+$ T cells. **j** Flow-cytometry analysis and quantification of immune checkpoint molecules expressed on tumor-infiltrating $CD4^+$ T cells.

Supplementary Table 1. Antibodies used for flow cytometry.

**FACS antibody**	**Source (#Cat)**	RRID
BD Horizon™ Fixable Viability Stain 450	BD (#562247)	RRID:AB_2869405
V450-conjugated anti-mouse CD11c	BD (#560521)	RRID:AB_1727423
V450-conjugated anti-mouse TCRb	BD (#560706)	RRID:AB_1727576
V450-conjugated anti-mouse Ly6G	BD (#560603)	RRID:AB_1727564
PE-conjugated anti-mouse Ly6C	Invitrogen (#12-5932-82)	RRID:AB_10804510
APC Cy7-conjugated anti-mouse Ly6C	BioLegend (#128026)	RRID:AB_10640120
FITC-conjugated anti-mouse CD45	BD (#561088)	RRID:AB_10562038
FITC-conjugated anti-mouse CD25	BD (#553072)	RRID:AB_394604
PE-conjugated anti-mouse MHC-II	BioLegend (#107608)	RRID:AB_313323
PE-conjugated anti-mouse CD80	BioLegend (#104708)	RRID:AB_313129
PE-conjugated anti-mouse CD86	BioLegend (#105008)	RRID:AB_313151
PE-conjugated anti-mouse CD163	BioLegend (#155308)	RRID:AB_2814062
PE-conjugated anti-mouse CD206	BioLegend (#141706)	RRID:AB_10895754
PeCy7-conjugated anti-mouse F4/80	BioLegend (#123114)	RRID:AB_893478
PerCP-Cy5.5-conjugated anti-mouse CD11b	BD (#550993)	RRID:AB_394002
PerCP-Cy5.5-conjugated anti-mouse CD4	BD (#550954)	RRID:AB_393977
PerCP-Cy5.5-conjugated anti-mouse CD8	BD (#561109)	RRID:AB_10563417
FITC-conjugated anti-mouse CD25	BD (#553072)	RRID:AB_394604
PE-Cy7-conjugated anti-mouse TCRb	BD (#560729)	RRID:AB_1937310
APC Cy7-conjugated anti-mouse IL-17a	BioLegend (#506940)	RRID:AB_2565781
PE-conjugated anti-mouse FoxP3	Invitrogen (#12-5773-82)	RRID:AB_465936
APC Cy7-conjugated anti-mouse CD107a	BioLegend (#121616)	RRID:AB_10643268
APC-conjugated anti-mouse granzyme B	Invitrogen (#17-8898-82)	RRID:AB_2688068
APC Cy7-conjugated anti-mouse FasL	Invitrogen (#47-5911-82)	RRID:AB_2762717
APC-conjugated anti-mouse IFN-g	BD (#554413)	RRID:AB_398551
PE-conjugated anti-mouse PD1	BIoLegend (#135206)	RRID:AB_1877231
APC-conjugated anti-mouse CTLA4	Invitrogen (#17-1522-82)	RRID:AB_2016700
APC-conjugated anti-mouse TIGIT	BioLegend (#142106)	RRID:AB_10962572
APC-conjugated anti-mouse LAG3	Invitrogen (#47-2231-82)	RRID:AB_2637323

Supplementary Table 2. Antibodies used for IHC and IF

**Antibody**	**Source (#Cat)**	RRID
Ki67	Abcam (#AB16667)	RRID:AB_302459
Active caspase3	Cell signaling (#9661S)	RRID:AB_2341188
F4/80	Santa cruz (#sc-377009)	RRID:AB_2927461
CD4	Santa cruz (#sc-13573)	RRID:AB_2108559
CD8	Santa cruz (#sc-18860)	RRID:AB_627184
IL-17	Santa cruz (#sc-374218)	RRID:AB_10988239
FoxP3	Santa cruz (#sc-53876)	RRID:AB_783444
CCL19	Bioss (#bs-2454R)	RRID:AB_10858015
CD163	Abcam (#AB87099)	RRID:AB_11154711
PD-L1	Abcam (#AB238697)	RRID:AB_2904145
PLD1	Santa cruz (#sc-28314)	RRID:AB_677324
PLD2	Santa cruz (#sc-515744)	-
CD24	Santa cruz (#sc-19585)	RRID:AB_626989
CD47	Abcam (#AB218810)	RRID:AB_3083705
Calreticulin	Abcam (#AB2907)	RRID:AB_303402
HMGB1	Abcam (#AB18256)	RRID:AB_444360
NF-kB (p65)	Abcam (#AB76311)	RRID:AB_2179019
Hoechst 33342	CiteAb (H3570)	RRID:AB_3675235
Goat anti-Mouse IgG, Alexa Fluor 488	CiteAb (A-11001)	RRID:AB_2534069
Goat anti-Mouse IgG, Alexa Fluor 594	CiteAb (A-11005)	RRID:AB_2534073

Supplementary Table 3. The primers for expression detection and quantification

Target gene	Primer sequences
Cxcl1	F: ACT GCA CCC AAA CCG AAG TC<br>R: TGG GGA CAC CTT TTA GCA TCT T
Ccl2	F: GCA TCT GCC CTA AGG TCT TCA<br>R: TGC TTG AGG TGG TTG TGG AA
Cxcl2	F: ATC CAG AGC TTG AGT GTG ACG<br>R: GTT AGC CTT GCC TTT GTT CAG
Ccl19	F: GGG GTG CTA ATG ATG CGG AA<br>R: CCT TAG TGT GGT GAA CAC AAC A
Ccl20	F: GCC TCT CGT ACA TAC AGA CGC<br>R: CCA GTT CTG CTT TGG ATC AGC
Vegfd	F: CCT ATT GAC ATG CTG TGG GAT<br>R: GTG GGT TCC TGG AGG TAA GAG
Tbx21	F: ATT GGT TGG AGA GGA AGC GG<br>R: GCA CCA GGT TCG TGA CTG TA
Rorc	F: ATC GTA GCC ACC AGT ACT CAG<br>R: GTT GTG GAA GAA CTC TGG GAA
FoxP3	F: TGG AAC CAC GGG CAC TAT CAC A<br>R: GAG GCT GCG TAT GAT CAG TTA TGC
Gzmb	F: GAC AAC ACT CTT GAC GCT GG<br>R: TGA TCT CCC CTG CCT TTG TCC
Prf1	F: GAT GTG AAC CCT AGG CCA GA<br>R: GGT TTT GTA CCA GGC GAG A
Fasl	F: CAC AAA TCT GTG GCT ACC G<br>R: GCC CAT ATC TGT CCA GTA G
actb	F: ATG CTC TCC CTC ACG CCA TC<br>R: CCA CGC TCG GTC AGG ATC TT

Supplementary Table 4. Antibodies used for western blotting

**Antibody**	**Source (#Cat)**	RRID
PD-L1	Abcam (#AB238697)	RRID:AB_2904145
Phospho-JAK2 (Tyr1007/1008)	Cell Signaling (#3771)	RRID:AB_330403
JAK2	Cell Signaling (#3230)	RRID:AB_2128522
Phospho-STAT1 (Tyr701)	Cell Signaling (#9167)	RRID:AB_561284
STAT1	Cell Signaling (#9172)	RRID:AB_2198300
Phospho-Stat3 (Tyr705)	NobusBio (NB100-80051)	RRID:AB_1110701
STAT3	Cell Signaling (#9139)	RRID:AB_331757
Phospho-PI3K p85 (Tyr458) /)/p55 (Tyr199)	Cell Signaling (#17366)	RRID:AB_2895293
PI3K p85	Cell Signaling (#4292)	RRID:AB_329869
Phospho-Akt (sSer473)	Cell Signaling (#9271)	RRID:AB_329825
Akt	Cell Signaling (#9272)	RRID:AB_329827
Phospho-IκB (Ser32/36)	Cell Signaling (#9246)	RRID:AB_2267145
IκB	Cell Signaling (#9247)	RRID:AB_2151426
CCL19	Bioss (#bs-2454R)	RRID:AB_10858015
🚲-Actin (AC-15)	Santa Cruz (#sc-69879)	RRID:AB_1119529
